# Mutual activation of glutamatergic mGlu_4_ and muscarinic M_4_ receptors reverses schizophrenia-related changes in rodents

**DOI:** 10.1007/s00213-018-4980-y

**Published:** 2018-07-27

**Authors:** Paulina Cieślik, Monika Woźniak, Jerri M. Rook, Mohammed N. Tantawy, P. Jeffrey Conn, Francine Acher, Krzysztof Tokarski, Magdalena Kusek, Andrzej Pilc, Joanna M. Wierońska

**Affiliations:** 1 Institute of Pharmacology, Polish Academy of Sciences, 12 Smętna St, 31-343 Kraków, Poland; 2 Department of Pharmacology, Vanderbilt University, Nashville, TN 37232 USA; 3 Vanderbilt Center for Neuroscience Drug Discovery, Vanderbilt University, Nashville, TN 37232 USA; 4 Laboratory of Pharmacological and Toxicological Chemistry and Biochemistry, UMR8601-CNRS, Paris Descartes University, Sorbonne Paris Cite, 45, rue des Saints-Peres, 75270 Paris Cedex 06, France; 5 Health Sciences Faculty, Institute of Public Health, Jagiellonian University Medical College, Kraków, Poland

**Keywords:** Schizophrenia, Animal model, Muscarinic receptor, Metabotropic glutamate receptor

## Abstract

**Rationale:**

Metabotropic glutamate receptors and muscarinic M_4_ receptors have been proposed as novel targets for various brain disorders, including schizophrenia. Both receptors are coupled to G_o/i_ proteins and are expressed in brain circuits that are important in schizophrenia. Therefore, their mutual activation may be an effective treatment and allow minimizing the doses of ligands required for optimal activity.

**Objectives:**

In the present studies, subactive doses of mGlu_4_ and M_4_ activators (LSP4-2022 and VU152100, respectively) were administered to investigate the mutual interaction between mGlu_4_ and M_4_ receptors in animal models of schizophrenia.

**Methods:**

The behavioral tests used were MK-801-induced hyperactivity, (±)-2.5-dimethoxy-4-iodoamphetamine hydrochloride (DOI)-induced head twitches, the modified forced swim test, and MK-801-induced disruptions of social interactions and novel object recognition. DOI-induced spontaneous excitatory postsynaptic currents (sEPSCs) in brain slices and positron emission tomography (PET) in were used to establish the ability of these compounds to modulate the glutamatergic and dopaminergic systems. Rotarod was used to assess putative adverse effects.

**Results:**

The mutual administration of subactive doses of LSP4-2022 and VU152100 exerted similar antipsychotic-like efficacy in animals as observed for active doses of both compounds, indicating their additive actions. VU152100 inhibited the DOI-induced frequency (but not amplitude) of sEPSCs in the frontal cortex, confirming presynaptic regulation of glutamate release. Both compounds reversed amphetamine-induced decrease in D_2_ receptor levels in the striatum, as measured with [^18^F]fallypride. The compounds did not induce any motor impartments when measured in rotarod test.

**Conclusions:**

Based on our results, the simultaneous activation of M_4_ and mGlu_4_ receptors is beneficial in reversing MK-801- and amphetamine-induced schizophrenia-related changes in animals.

**Electronic supplementary material:**

The online version of this article (10.1007/s00213-018-4980-y) contains supplementary material, which is available to authorized users.

## Introduction

Schizophrenia is a brain disorder that affects approximately 1% of the human population. The disease is less common than other psychiatric disorders, such as depression or anxiety (Global Burden of Disease Study 2013), but is considered as one of the most severe mental health disorders. Schizophrenia is estimated to cause 1% of worldwide disability adjusted life years (DALYs) due to long-term unemployment, poverty, and homelessness (Ormel et al. 2008; Rössler et al. 2005). Unfortunately, only approximately 20% of patients with schizophrenia are effectively treated with current medications. The most treatment-resistant symptoms of schizophrenia are negative and cognitive symptoms, which simultaneously have a greater contribution to a poor quality of life and functional disability than the positive symptoms (Velligan et al. 2009; Kane and Mayerhoff 1989). The efficacy of typical neuroleptics towards positive symptoms of schizophrenia is relatively good, although typical antipsychotics induce a variety of adverse effects due to the blockade of D_2_ receptors in the striatum, including extrapyramidal motor effects (Corripio et al. 2012; Bo et al. 2016).

Antipsychotic drug discovery constitutes the main field of interest of many research groups, and many potential antipsychotic drug targets have been developed. Metabotropic glutamate receptors (mGlu), which were discovered in 1985, constitute one of such targets (Sladeczek et al. 1985; Schoepp et al. 1999; Nicoletti et al. 2015). At least three mGlu receptors subtypes are considered when developing antipsychotic treatments, including mGlu_2_, mGlu_4_, and mGlu_5_ receptors. The activation of these receptors with agonists or positive allosteric modulators (PAMs) induces antipsychotic-like effects in variety of animal models of schizophrenia (Conn et al. 2009b, c; Wierońska et al. 2016; Poels et al. 2014; Muguruza et al. 2016; Ellaithy et al. 2015; Lindsley and Stauffer 2013; Hashimoto et al. 2013).

The muscarinic acetylcholine (ACh) receptor ligands represent another emerging approach in antipsychotic drug discovery. M_1_ and M_4_ are the most heavily expressed in the central nervous system (CNS) and represent attractive therapeutic targets for brain disorders, including schizophrenia (Bymaster et al. 2002; Messer 2002; Raedler et al. 2007). Although the expression of M_4_ receptors was also observed in peripheral tissues (lungs and enteric neurons), the adverse effects of cholinergic agents are thought to be primarily due to activation of peripheral M_2_ and M_3_ mAChRs (Bymaster et al. 2003a, b).

A number of selective M_4_ receptor ligands were synthesized and their efficacy in treating animal models of several CNS disorders, including schizophrenia, was proposed (Byun et al. 2014; Brady et al. 2008; Dencker et al. 2012). Specific modulators of the M_4_ receptor, VU152100, VU0152099, or LY2033298, reversed the amphetamine-induced hyperlocomotion in rats (Brady et al. 2008; Suratman et al. 2011), were active in self-administration and cocaine-induced hyperactivity, and enhanced associative learning in rodents (Byun et al. 2014; Dencker et al. 2012; Brady et al. 2008; Bubser et al. 2014).

In the present studies, the synergic antipsychotic action of mGlu_4_ and M_4_ receptor activation was investigated. The basic assumption of the study was to establish if the administration of subeffective doses of the ligands of those receptors would exert antipsychotic-like activity without inducing adverse effects typical for standard dopamine-based antipsychotics. Such simultaneous action of the combined treatment was reported previously for subeffective doses of mGlu_4_-5-HT_1A_ receptor ligands (Wierońska et al. 2013, 2015). In addition, studies were undertaken with the combined administration of mGlu_4_ and GABA_B_ activators, but the efficacy of subeffective doses of the combination of these ligands was not evident in the models of negative and cognitive symptoms of schizophrenia, although the ligands exerted antipsychotic efficacy when active doses of each compound were administered alone (Wierońska et al. 2015; Woźniak et al. 2016).

Behavioral, neurochemical, and brain imaging techniques were used to assess the putative interaction between M_4_ and mGlu_4_ receptors. Ligands with known activity profiles such as VU152100 and LSP4-2022 were used. The activity of the compounds was tested in MK-801- and amphetamine-induced hyperactivity tests, DOI-induced head twitches, social interactions, the modified forced swim test, and novel object recognition tests. The activity of VU152100 on DOI-induced spontaneous excitatory postsynaptic currents (sEPSCs) in the brain slices from frontal cortex was examined. Finally, positron emission tomography (PET) imaging was used to establish whether the drugs were able to reverse the amphetamine-induced decrease in D_2_ receptor levels in the striatum. Rotarod was used to establish if the compounds induce any adverse effects, alone or in the combinations.

## Materials and methods

### Animals and housing

Male Albino Swiss mice (18–20 g Charles River Laboratory, Germany) were used in behavioral tests and electrophysiology (see details below). Male Wistar rats (250–300 g, Envigo, Inc., Indianapolis, USA) were used in PET imaging and amphetamine-induced hyperactivity. The animals were housed 4 (rats) and 10 (mice) in standard laboratory cages under a 12:12 light–dark cycle in a room with a temperature of 19–21 °C, 50–60% humidity, and had free access to food and water. All compounds were administered in a volume of 10 ml/kg when given to mice and 1 ml/kg when injected into rats. The experimental assessments were performed by an observer who was blinded to the treatment. The procedures were conducted in accordance with the European Communities Council Directive of 22 September 2010 (2010/63/EU) and Polish legislation acts concerning animal experimentation. The experiments were approved by II Local Ethics Committee in Krakow by the Institute of Pharmacology, Polish Academy of Sciences in Krakow (no. 16/2017; 17/2017) and National Institutes of Health Animal Care and Use Committee approved by the Institutional Animal Care and Use Committee in the USA (microPET, M/15/209 and M/15/206).

### Drugs

The following drugs were used: LSP4-2022 (mGlu_4_ receptor agonist, [(3S)-3-Amino-3-carboxy)propyl][(4-(carboxymethoxy)phenyl)hydroxymethyl]phosphinic acid) was synthesized in Francine Acher’s laboratory. The compound is a derivative of its precursor, LSP1-2111, and was profiled as the best currently available orthosteric agonist of mGlu_4_ receptors (Goudet et al. 2012; Cajina et al. 2013). No activity at muscarinic receptors (M_1_–M_5_) was detected in functional studies, which were performed by DiscoverX (Table 1). The compound was dissolved in saline. The administration schedule for LSP4-2022 was based on our previous studies (Woźniak et al. 2016, 2017). VU152100 (3-Amino-*N*-(4-methoxybenzyl)-4,6-dimethylthieno[2,3-*b*]pyridine carboxamide, Tocris Bioscience, Bristol, UK) was dissolved in 10% Tween 80. Dosing of the compound was partially based on the results from previous studies (Byun et al. 2014), as well as on our own dose dependence studies. In the behavioral experiments, subthreshold doses for LSP4-2022 and VU0152100 were used in order to examine the antipsychotic action of simultaneous activation of mGlu_4_ and M_4_ receptors. For clear information which dose was subtreshold for each compound, please see Table 2. Both compounds were administered i.p, 30 min (VU152100) or 45 min (LSP4-2022) before DOI, MK-801, amphetamine, or appropriate vehicle administration. MK-801 ((5*R*,10*S*)-(-)-5-methyl-10,11-dihydro-5*H*-dibenzo[*a*,*d*]cylcohepten-5,10-imine maleate) and DOI (4-iodo-2,5-dimethoxy-α-methylbenzeneethanamine hydrochloride) (Tocris Bioscience, Bristol, UK) were dissolved in 0.9% NaCl and injected i.p. Different doses of MK-801 were applied to obtain optimal effects in each test, which is consistent with our previous studies (Wierońska et al. 2012, 2013; Woźniak et al. 2017) and the studies of other research groups (Geyer and Ellenbroek 2003). Amphetamine ((+)-α-methylphenethylamine hemisulfate salt, Sigma-Aldrich) was dissolved in 0.9% saline and administered s.c. Risperidone (3-[2-[4-(6-fluoro-1,2-benzisoxazol-3-yl)-1-piperidinyl]ethyl]-6,7,8,9-tetrahydro-2-methyl-4*H*-pyrido[1,2-*a*]pyrimidin-4-one, Tocris Bioscience, Bristol, UK) and haloperidol (4-[4-(4-chlorophenyl)-4-hydroxy-1-piperidinyl]-1-(4-fluorophenyl)-1-butanone, WZF Polfa S.A.) were dissolved in 0.2% Tween 80 and administered i.p, 30 min before experiments (based on preliminary experiments and our previous studies Sławińska et al. 2013). All animals that were not treated with drugs (control groups) received appropriate vehicles.

**Table 1:** LSP4-2022 activity with the GPCR biosensor assays

Compound	Assay name	Assay format	Assay target	Concentration (μM)	Average value	Standard deviation	% efficacy
LSP4-2022	Arrestin	Agonist	M1	1	828,240	28,510	− 2.5
LSP4-2022	Arrestin	Agonist	M1	10	779,100	21,976	− 6
LSP4-2022	Arrestin	Agonist	M1	25	793,800	5147	− 5
LSP4-2022	Arrestin	Agonist	M1	50	819,700	31,876	− 3.1
LSP4-2022	Arrestin	Agonist	M1	100	807,800	10,295	− 4
LSP4-2022	Arrestin	Agonist	M2	1	71,260	3761	2
LSP4-2022	Arrestin	Agonist	M2	10	63,560	5939	1.1
LSP4-2022	Arrestin	Agonist	M2	25	65,380	11,681	1.3
LSP4-2022	Arrestin	Agonist	M2	50	59,360	1583	0.6
LSP4-2022	Arrestin	Agonist	M2	100	57,820	4553	0.4
LSP4-2022	Arrestin	Agonist	M3	1	31,220	3365	− 2.8
LSP4-2022	Arrestin	Agonist	M3	10	29,960	2771	− 3.1
LSP4-2022	Arrestin	Agonist	M3	25	30,660	198	− 2.9
LSP4-2022	Arrestin	Agonist	M3	50	31,220	1781	− 2.8
LSP4-2022	Arrestin	Agonist	M3	100	33,600	396	− 2.1
LSP4-2022	Arrestin	Agonist	M4	1	15,540	1385	− 7.7
LSP4-2022	Arrestin	Agonist	M4	10	16,800	3563	− 2.7
LSP4-2022	Arrestin	Agonist	M4	25	16,100	594	− 5.5
LSP4-2022	Arrestin	Agonist	M4	50	16,520	396	− 3.8
LSP4-2022	Arrestin	Agonist	M4	100	14,980	594	− 9.8
LSP4-2022	Arrestin	Agonist	M5	1	1,890,700	14,057	− 8.1
LSP4-2022	Arrestin	Agonist	M5	10	1,942,780	31,480	− 6.1
LSP4-2022	Arrestin	Agonist	M5	25	1,885,240	96,223	− 8.3
LSP4-2022	Arrestin	Agonist	M5	50	1,902,320	90,679	− 7.6
LSP4-2022	Arrestin	Agonist	M5	100	1,896,720	4751	− 7.9

LSP4-2022 was tested in agonist mode, and data was normalized to the maximal and minimal response observed in the presence of control ligand (acetylcholine) and vehicle

**Table 2:** The subthreshold doses of LSP4-2022 and VU152100 administered in each test

Test	LSP4-2022	VU152100
MK-801-induced hyperactivity	0.1 mg/kg	5 mg/kg
DOI-induced head twitches	0.5 mg/kg	1 mg/kg
Modified forced swim test	0.1 mg/kg	0.1 mg/kg
Social interaction	0.1 and 0.5 mg/kg	2 and 5 mg/kg
Novel object recognition	1 mg/kg	0.25 mg/kg
Amphetamine-induced hyperactivity and PET studies (rats)	0.1 mg/kg	5 mg/kg

### MK-801-induced hyperactivity in mice

Locomotor activity was recorded in locomotor activity cages (according to Rorick-Kehn et al. 2007; Wierońska et al. 2012, 2013). The locomotor activity was recorded individually for each animal in OPTO-M3 locomotor activity cages (Columbus Instrument) linked online to a compatible PC. Each cage (13 cm × 23 cm × 15 cm) was surrounded with an array of photocell beams. Interruptions of these photobeams resulted in horizontal activity defined as ambulation counts. The mice were individually placed into actometers for an acclimation period of 30 min. Then, VU152100 (5 mg/kg) and LSP4-2022 (0.1 mg/kg) were administered. MK-801 was i.p. administered at a dose of 0.35 mg/kg and locomotor activity was measured for 60 min immediately after the injection. All groups were compared with the MK-801 control group. The experiment also included a control group that was not treated with MK-801.

### Head twitch test

The experiment was performed according to the methods reported by Wierońska et al. (2012, 2013). Each animal was transferred to a 12 (diameter) × 20 cm (height) glass cage lined with sawdust 30 min before the experiment. The head twitches of the mice were induced by an i.p. injection of DOI (2.5 mg/kg). VU152100 was administered at the doses of 0.5, 1, 2, and 10 mg/kg 30 min before the DOI injection. In the combined administration, VU152100 was administered at the dose 1 mg/kg, while LSP4-2022 was administered at the dose of 0.25 mg/kg. The number of head twitches was counted during a 20-min session immediately after DOI administration.

### Social interaction test

The social interaction test was performed according to a previously described method (Oh et al. 2013; de Moura Linck et al. 2008; Woźniak et al. 2017). The body weights of the paired mice were matched to within a 10% difference. Both adaptation (2 days, 10 min of free exploration) and the subsequent test were conducted in black plastic boxes (50 × 30 × 35 cm) illuminated with the light intensity of 335 lx. The social interactions between two mice were determined based on the total time spent participating in social behaviors, such as sniffing, genital investigation, chasing, and fighting each other, during a 10-min test. Each dose of VU152100 (0.5, 2, and 5 mg/kg) was co-administered with subtreshold (0.1 and 0.5 mg/kg) or active (1 mg/kg) dose of LSP4-2022. The doses of LSP4-2022 were chosen according to our previous studies (Woźniak et al. 2017). MK-801 (0.3 mg/kg) was administered 30 min before the test. Control experiments with animals that did not receive MK-801 were conducted to determine whether the drugs had any influence on social behavior when administered alone.

### Modified forced swim test

The modified forced swim test was performed according to the method introduced by Noda (Noda et al. 1995, 1997), Wierońska et al. (2015), and Woźniak et al. (2016, 2017). The swim tests were performed in a glass cylinder (height, 20 cm; internal diameter, 15 cm) containing 11 cm of water maintained at 23–26 °C. After the acclimation period, the animals underwent the first swim test, where the immobility time was measured during a 3-min period (*T*_1_). On the next day, chronic (13 days) MK-801 administration (0.4 mg/kg, i.p.) was started. After a 1-day break, on the 15th day of experiment, the second swim session was performed and the immobility time during 3-min test was measured again (*T*_2_). The *T*_2_ − *T*_1_ difference was reported as the result of the experiment. Drugs were administered acutely before the *T*_2_ session. VU152100 was administered at the doses of 0.5, 1, and 2 mg/kg (30 min before the test), and then the subthreshold dose of the compound (0.1 mg/kg) was co-administered with subthreshold dose of LSP4-2022 (0.1 mg/kg, 45 min before the test).

### Novel object recognition

The method was performed as described by Nilsson et al. (2007) and Woźniak et al. (2017). The animals were trained and tested in a black plastic rectangular open field (50 × 30 × 35 cm). The open field was placed in a dark room and was illuminated with only the light intensity of 335 lx. After 2 days of adaptation (10 min of free exploration), the animals were placed in the apparatus on the experimental day and allowed to explore two identical objects (a red, glass cylinder, 6.5 cm in diameter, 4.5 cm high) for 10 min. For the retention trial (*T*_2_) that was conducted 1 h later, one of the objects presented in *T*_1_ was replaced with a novel object (a transparent glass elongated sphere-like object with an orange cap). The duration of exploration of each object (i.e., sitting in close proximity to the objects or sniffing or touching them) during 5 min was video-recorded and measured separately by a trained observer. The results were calculated as recognition index, defined as (*T*_novel_ − *T*_familial_ / *T*_familial_ + *T*_novel_) × 100. All drugs were administered before the training (*T*_1_) session. MK-801 (0.3 mg/kg) was administered 30 min before the session. Each dose of VU152100 (0.25, 0.5, and 1 mg/kg) was co-administered with subtreshold (0.1 and 0.5 mg/kg) or active (1 mg/kg) dose of LSP4-2022. The doses of LSP4-2022 were chosen according to our previous studies (Woźniak et al. 2017). Control experiments with animals that did not receive MK-801 were conducted to determine whether the drugs had any influence on social behavior when administered alone.

### DOI-induced sEPSCs

Albino Swiss mice were decapitated; their frontal cortices were dissected and cut into slices (420 μm thick) in the frontal plane using a vibrating microtome. Slices were submerged in artificial cerebrospinal fluid (ACSF) consisting of (in mM) 126 NaCl, 4 KCl, 2.5 CaCl_2_, 1.3 MgSO_4_, 1.25 KH_2_PO_4_, 26 NaHCO_3_, and 10 glucose, bubbled with 95% O_2_/5% CO_2_, pH = 7.4. A single slice was transferred to the recording chamber (volume 1 ml) and superfused with warmed (32 °C) ACSF at 2 ml/min. Individual neurons were visualized using an upright microscope (Zeiss Axioskop 2FS) equipped with a long-range water immersion objective (×40) and an infrared camera. Recording micropipettes were pulled on a Flaming-Brown puller (P-87; Sutter Instruments, Novato, CA, USA) and had a resistance of 6–8 MΩ. Microelectrodes were filled with (in mM) 130 K-gluconate, 5 KCl, 0.3 CaCl_2_, 2 MgCl_2_, 1 EGTA, 10 HEPES, 5 Na_2_-ATP, 0.4 and Na-GTP, with osmolarity of 290 mOsm and pH = 7.2. Whole-cell recordings were obtained from layer V pyramidal cells in the cortex. After confirming the electrophysiological characteristics of the neurons in current clamp mode, cells were voltage-clamped at − 76 mV and sEPSCs were recorded. Signals were acquired using the SEC 05 L amplifier (NPI, Germany) and digitized using the Digidata 1322 interface (Molecular Devices, Sunnyvale, CA, USA). Drugs stored as concentrated stocks were diluted in ACSF just before the experiment and applied to the superfusate. After achieving a stable control recording for at least 15 min, DOI (10 μM) was applied for 15 min and sEPSCs were recorded (8 min). Next, DOI was applied concurrently with VU152100, LSP4-2022, and VU152100/LSP4-2022 for 15 min and sEPSCs were again recorded. The measured parameters were the frequency and amplitude of sEPSCs. The data were analyzed off-line using the Mini Analysis program (Synaptosoft Inc., ver. 6.0.3).

### Amphetamine-induced hyperactivity in rats

Rats were habituated to the locomotor activity cages for 30 min. The locomotor activity was recorded individually for each animal in Opto-Varimex cages (Columbus Instruments, Columbus, OH, USA) connected to a compatible IBM-PC. Each chamber (43 cm × 43 cm × 21 cm) was made of transparent acrylic plastic (all six sides), equipped with a 220 lx house light, and was placed in a light- and soundproof wooden cubicle. The corner brackets were made of stainless steel. Each cage was surrounded by a 15 × 15 array of photocell beams located 3 cm from the floor surface. Interruptions of these photobeams resulted in horizontal activity defined as ambulation counts. The rats were injected with LSP4-2022 (0.1 and 2 mg/kg), VU152100 (2.5, 5, and 15 mg/kg), and with combined treatment of VU152100 (5 mg/kg) and LSP4-2022 (0.1 mg/kg). Amphetamine was administered s.c. at a 1-mg/kg dose and the locomotor activity was measured for 60 min immediately after the injection.

### MicroPET imaging

Rats were imaged according to the procedures outlined in a previous work (Tantawy et al. 2009, 2011). Briefly, rats were anesthetized with < 2% isoflurane and injected with ~ 13 MBq/0.2 ml [^18^F]fallypride, followed by a 0.1-ml of saline via a tail vein catheter. Rats were under anesthesia for less than 10 min. Rats were then returned to their cages and fed ad libitum. Rats returned to full activity within 10–20 min after isoflurane had been removed. Fifty minutes later, rats were anesthetized with < 2% isoflurane and positioned in an Inveon microPET/CT (Siemens, Knoxville TN). A CT scan was initiated with an x-ray beam intensity of 25 mAs and an x-ray peak voltage of 80 kVp, followed by a 60-min dynamic PET scan acquisition. The PET scans always started at 60 min after radiotracer administration. The 60-min dynamic acquisition was divided into six frames of a 600-s duration each. All datasets were reconstructed using the OSEM-2D algorithm into 128 × 128 × 95 slices with a voxel size of 0.095 × 0.095 × 0.08 cm^3^, after correcting for scatter and attenuation. The resulting images were manually co-registered to an MRI brain template (Rubins et al. 2003; Schweinhardt et al. 2003) using the medical imaging analysis tool AMIDE software (Loening and Gambhir 2003). Anatomical volumetric regions-of-interest (ROIs) were drawn around the left striatum, right striatum, and cerebellum. The radiotracer concentrations within the ROIs were used to estimate the modified distribution volume ratio (DVR′) (Tantawy et al. 2009), where the cerebellum, which expresses few or no D_2_ receptors, was used as the reference tissue. Percent occupancy was calculated as: percent occupancy = ((DVR′_Tvehicle_ − DVR′_Ttreatment_) / DVR′_Tvehicle_) × 100.

Rats were injected with amphetamine (1 mg/kg, s.c.) 15 min prior to the administration of [^18^F]fallypride. The investigated compounds, LSP4-2022 and VU152100, were administered 45 and 30 min, respectively, before amphetamine administration. Four groups of rats were tested: AMPH+LSP4-2022 (2 mg/kg), AMPH+LSP4-2022 (0.1 mg/kg), AMPH+VU152100 (15 mg/kg), and AMPH+VU152100 (5 mg/kg).

### Rotarod test

The rotarod test was performed as described by Vogel et al. (2008) with small modifications. The animals were trained for three consecutive days at the speed of 18 rpm, one session per day for 3 min. If a mouse fell during the habituation period, it was placed back on the apparatus. On the following day, the test trial was performed. After the mice were placed on the apparatus (Mouse Rotarod NG, UGO BASILE S.R.L.) moving at the speed of 12 rpm, the accelerating mode was started (maximum speed 24 rpm). The latency to fall was measured during 3-min test session. Mice were injected with VU152100 (0.5; 5 mg/kg), LSP4-2022 (0.1; 2 and 5 mg/kg), risperidone (0.1; 0.5 mg/kg), or haloperidol (0.2; 1 mg/kg). Then, different combinations of subtreshold doses of VU152100 with LSP4-2022 were administered, as well as subtreshold dose of VU152100 (0.5 mg/kg) and LSP4-2022 (0.1 mg/kg) were co-administered with two doses of haloperidol or risperidone. Mice were administered with the investigated compounds 30 min before the test, except LSP4-2022, which was administered 45 min before the test.

### Statistical analysis

The data are presented as means ± SEM. Statistical analyses of the data were performed using the Statistica 10 package (StatSoft Inc., OK, USA). One-way ANOVA followed by the Newman-Keuls post hoc analysis was used in dose dependence studies, and two-way ANOVA followed by the Newman-Keuls post hoc comparison test was used for the interaction studies. Student’s *T* test was used to determine the significance of the results obtained in electrophysiological recordings. *P* values < 0.05 were considered statistically significant.

## Results

### Locomotor activity studies

**Fig. 1:**
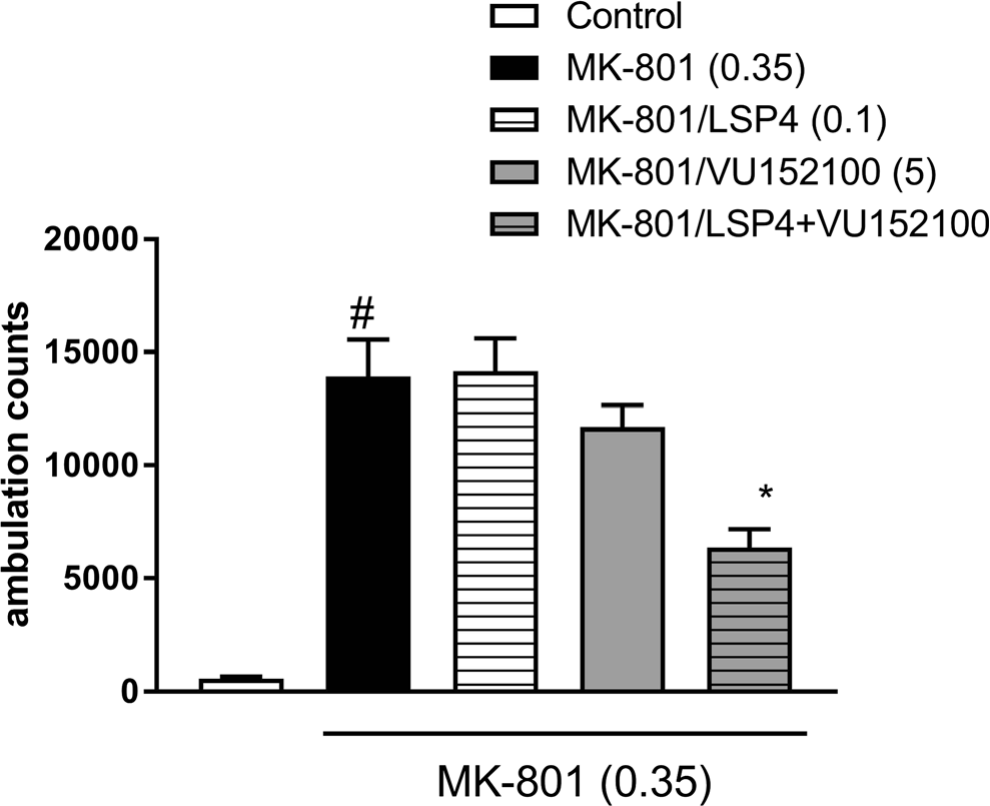
Effects of VU152100 and LSP4-2022 on MK-801-induced hyperactivity in mice that had been habituated to locomotor activity cages. LSP4 and VU152100 were administered 45 and 30 min, respectively, before MK-801 administration. Locomotor activity was measured for 60 min immediately after the MK-801 injection. Doses in milligrams per kilogram are indicated in parentheses. Data are presented as means ± SEM. Two-way ANOVA revealed a statistically significant interaction [*F*_(1.35)_ = 4.69; *P* < 0.03]. ^#^*P* < 0.001 compared with the control group. *P < 0.05 compared with the MK-801-treated group. Number of animals in each group *n* = 10

At a dose of 0.35 mg/kg, MK-801 induced a typical increase in locomotor activity (*P* < 0.001). The administration of subeffective doses of VU152100 (5 mg/kg) and LSP4-2022 (0.1 mg/kg) did not influence MK-801-induced hyperlocomotion. The co-administration of both compounds at the doses indicated above resulted in a statistically significant reversal of MK-801-induced hyperactivity (Fig. 1). The administration of the combination without MK-801 did not have any influence on locomotor activity (Table 3).

**Table 3:** Control experiments for the most active doses of VU152100 and combinations of LSP4-2022+VU152100 in behavioral studies

	Locomotor activity of habituated animals (ambulation counts)	Social interaction (time of interaction in s)	Novel object recognition (recognition index)	Modified forced swim test (ambulation counts)
Control	1100 ± 100	18.1 ± 1.9	0.35 ± 0.03	1308 ± 112
VU152100 (5 mg/kg)	1050 ± 142 ns	19.14 ± 1.9 n.s		
VU152100 (2 mg/kg) + LSP4 (0.1 mg/kg)		19.43 ± 1.02 n.s		
VU152100 (1 mg/kg)			0.28 ± 0.02	
VU152100 (0.25 mg/kg) + LSP4 (0.1 mg/kg)			0.36 ± 0.02	
VU152100 (2 mg/kg)				1262 ± 157 ns
VU152100 (0.1 mg/kg) + LSP4 (0.1 mg/kg)				1203 ± 110 ns
VU152100 (5 mg/kg) + LSP4 (0.1 mg/kg)	993 ± 95 ns			

The table shows the effects of the two activators in the absence of MK-801 for the corresponding behavioral tests. Number of animals in each group *n* = 8

### DOI-induced head twitches

**Fig. 2:**
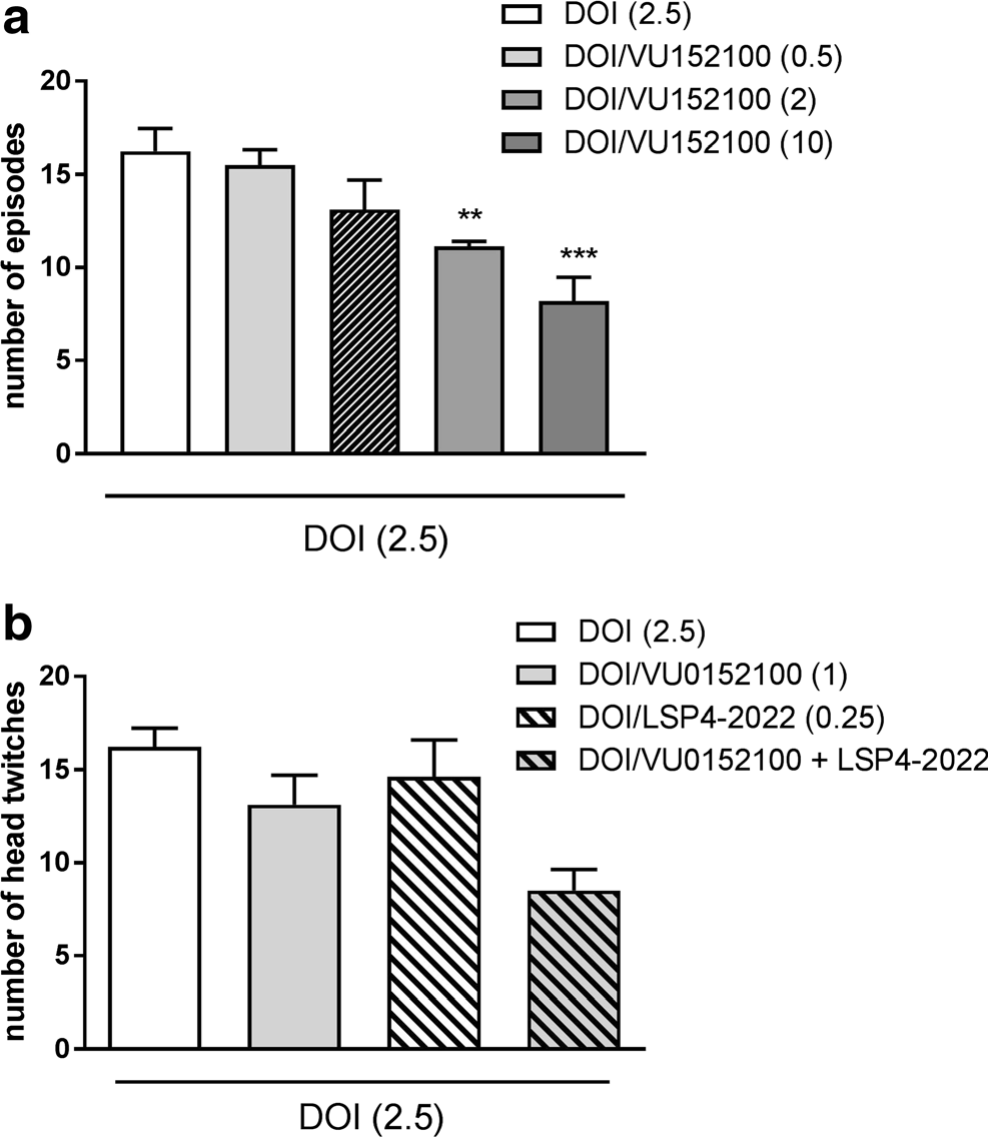
Effects of VU152100 (**a**) and the combined administration of VU152100 and LSP4-2022 (**b**, **c**) on DOI-induced head twitches. LSP4 and VU152100 were administered 45 and 30 min, respectively, before DOI administration. The number of head twitches was measured for 20 min immediately after DOI administration. Doses in milligrams per kilogram are indicated in parentheses. Data are presented as means ± SEM. One-way ANOVA [*F*_(4.31)_ = 7.05; *P* < 0.0004], **P < 0.01 and ***P < 0.005 compared with the control group. Number of animals in each group *n* = 7. The effect of LSP4-2022/VU152100 (**c**) did not reach statistical significance [*F*_(1.29)_ = 1.07, *P* = 0.3]

At the doses of 2 and 10 mg/kg, VU152100 induced a significant reduction of DOI-induced head twitches, while it was ineffective at the doses of 0.5 and 1 mg/kg (Fig. 2a). The co-administration of subthreshold doses of both compounds (VU152100 1 mg/kg and LSP4-2022 0.25 mg/kg) partially antagonized DOI-induced effect, but the effect was not statistically significant (Fig. 2b).

### Social interaction

**Fig. 3:**
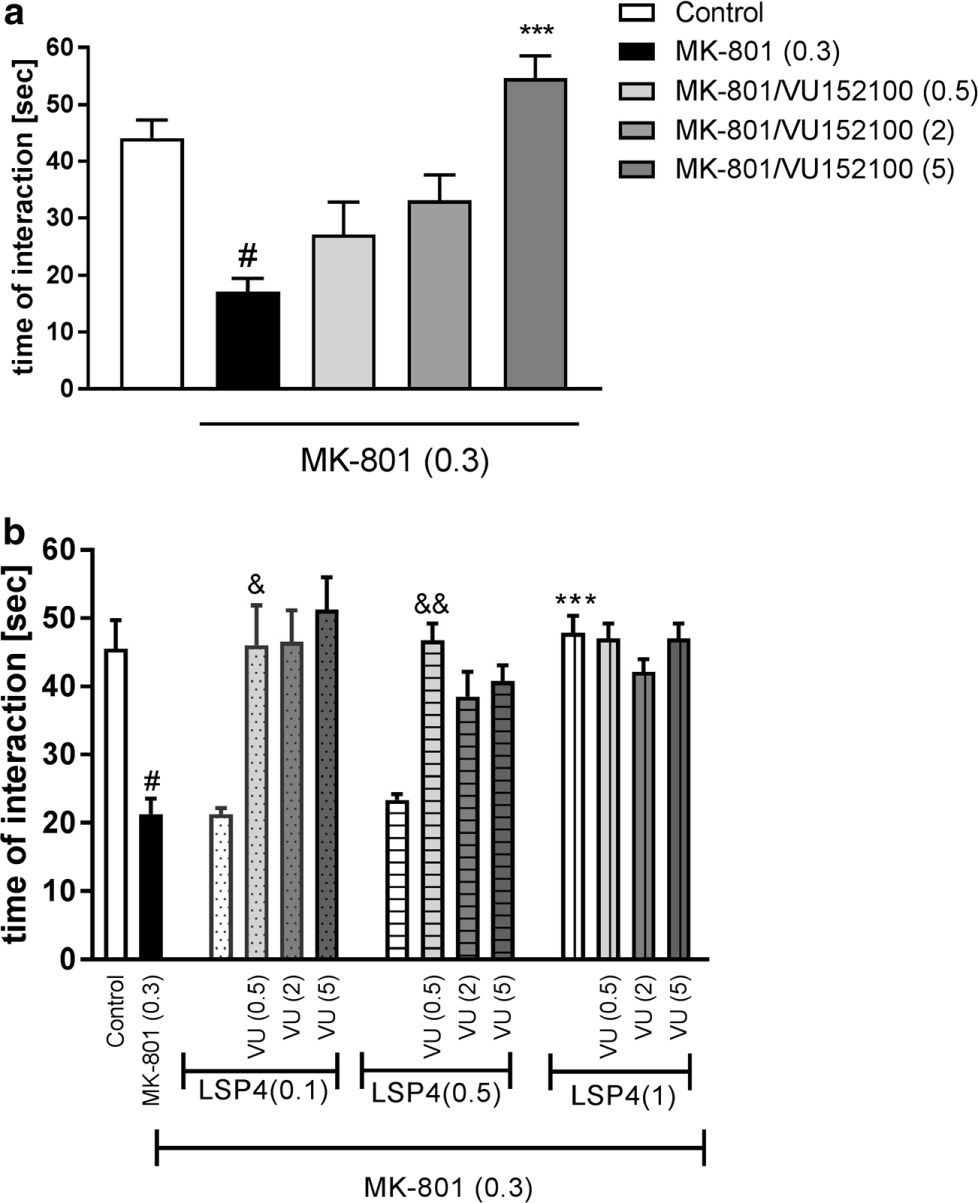
Effects of VU152100 (VU) and LSP4-2022 (LSP4) on MK-801-induced social interaction deficits. The time spent in social interactions was measured. **a** Effects of VU152100 administration and (**b**) effects of the combined administration of all three doses of VU152100 with LSP4 at subthreshold (0.1 and 0.5) and active (1) doses are shown. LSP4 and VU152100 were administered 45 and 30 min, respectively, before MK-801 administration. Doses in milligrams per kilogram are indicated in parentheses. Data are presented as means ± SEM. One-way ANOVA [*F*_(4.27)_ = 12.18; *P* < 0.01 and *F*_(4.27)_ = 11.39; *P* < 0.01] (**a**) and two-way ANOVA analysis [^&^*F*_(1.27)_ = 5.08; *P* < 0.03 (versus LSP4 0.1 mg/kg and VU 0.5 mg/kg) and ^&&^ *F*_(1.27)_ = 27,05; *P* < 0.00005 (versus LSP4 0.5 mg/kg and VU 0.5 mg/kg)] (**b**). ^#^*P* < 0.01 compared with the control group, ***P < 0.0001 compared with the MK-801-treated group, ^&^*P* < 0.01 compared to LSP4 (0.1 mg/kg) and VU (0.5 mg/kg) treated groups, ^&&^*P* < 0.0001 compared to LSP4 (0.5 mg/kg) and VU (0.5 mg/kg) treated groups. Number of animals in group varied *n* = 8–10

At a dose of 0.3 mg/kg, MK-801 induced a disruption of social behaviors, as observed in the decrease in the duration of social contacts and in the number of episodes. At a dose of 5 mg/kg, VU152100 clearly reversed the MK-801-induced effects on both the time of interaction and the number of episodes. The administration of 0.5 and 2 mg/kg doses of VU152100 was ineffective (Fig. 3a).

The co-administration of subthreshold doses of both VU152100 (2 mg/kg) and LSP4-2022 (0.1 or 0.5 mg/kg) totally reversed the effect of MK-801 in a way comparable to the effect of the most active dose of VU152100 alone (Fig. 3b). The co-administration of VU152100 at the higher dose 2 mg/kg with subthreshold doses of LSP4-2022 (0.1 and 0.5 mg/kg) also reversed the action of MK-801 to the level achieved by the administration of the most active dose of VU152100 or LSP4-2022 alone. The action of the most active dose of VU152100 (5 mg/kg) was not enhanced when co-administered with LSP4-2022 at all three doses (Fig. 3b).

Neither VU152100 nor the combination of subtreshold doses of VU152100 with LSP4-2022 changed the behavior of animals when administered in the absence of MK-801 (Table 3).

### Modified forced swim test

**Fig. 4:**
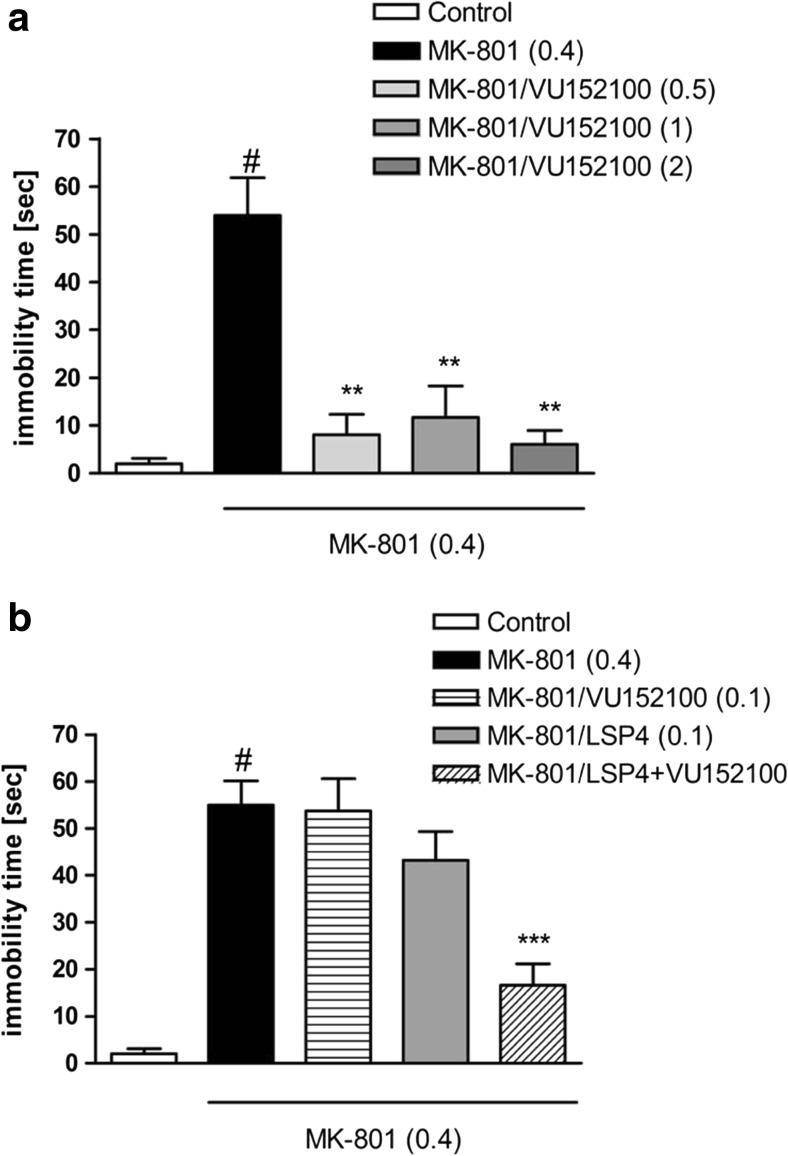
Effects of VU152100 (**a**) and the combined administration of VU152100 with LSP4 (**b**) on the immobility time in the modified forced swim test after chronic administration (13 days) of MK-801. Doses in milligrams per kilogram are indicated in parentheses. Data are presented as means ± SEM. ^#^One-way ANOVA [*F*_(3.36)_ = 15.72; *P* < 0.001] (**a**) and two-way ANOVA of the effects [*F*_(1.36)_ = 4.99; *P* < 0.05], ^#^*P* < 0.01 compared with the control group, **P < 0.02 and ***P < 0.001 compared with the MK-801-treated group. Number of animals in each group *n* = 10

Chronic administration of MK-801 increased the immobility time in *T*_2_ session. The results are shown as a difference in the immobility time between *T*_2_ and *T*_1_ sessions (*P* < 0.01). VU152100 reversed this MK-801-induced effect at doses of 0.5, 1, and 2 mg/kg (Fig. 4a). The co-administration of the ineffective dose of the compound (0.1 mg/kg) together with a subthreshold dose of LSP4-2022 (0.1 mg/kg) displayed the same effect as the active doses of VU152100 (Fig. 4b). The spontaneous locomotor activity was not changed after the MK-801 administration or after the VU152100 or VU152100+LSP4-2022 administration (Table 3).

### Novel object recognition test

**Fig. 5:**
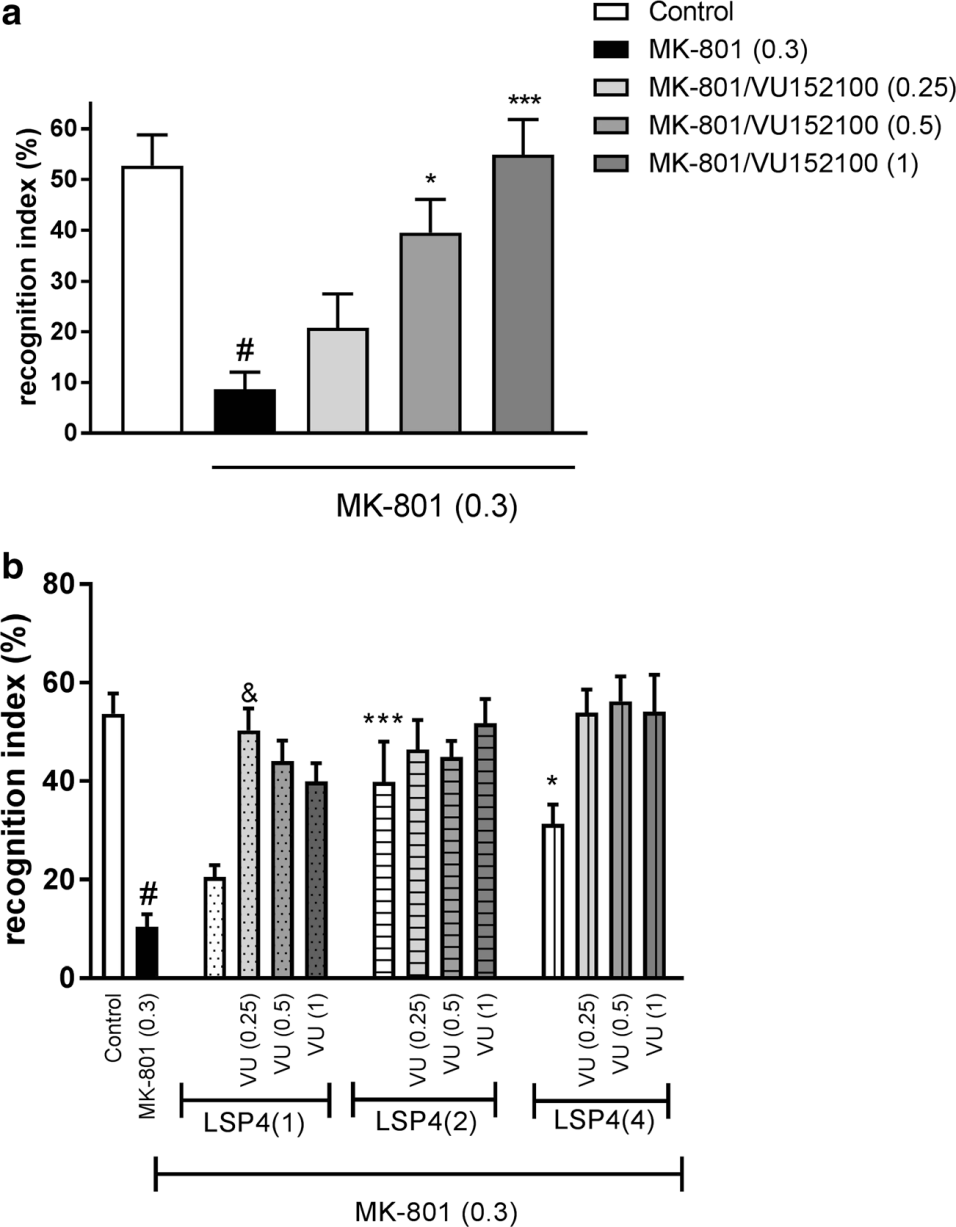
Effects of VU152100 (**a**), the combined administration of VU152100 with LSP4 (**b** and **c**) and the administration of VU152100 to mGlu_4_ KO mice (**d**) on MK-801-induced deficits in the NOR test. LSP4 and VU152100 were administered 45 and 30 min, respectively, before MK-801 administration. Doses in milligrams per kilogram are indicated in parentheses. Data are presented as means ± SEM. One-way ANOVA [*F*_(3.26)_ = 9.62; *P* < 0.01] (**a**) and two-way ANOVA of main effects [*F*_(1.29)_ = 5.17; *P* < 0.0002] (**b**). ^#^*P* < 0.001 compared with the control group, *P < 0.05, ***P < 0.001 compared with the MK-801-treated group, ^&^*P* < 0.05 compared with LSP4 (1 mg/kg) and VU (0.25 mg/kg) treated groups. Number of animals in each group *n* = 8–10

MK-801 induced a disruption in the novel object recognition behavior, as measured by the recognition index (*P* < 0.001). VU152100 reversed this MK-801-induced effect at doses of 0.5 and 1 mg/kg. The administration of a 0.25-mg/kg dose of the compound was ineffective (Fig. 5a).

The co-administration of subthreshold doses of VU152100 (0.25 mg/kg) and LSP4-2022 (1 mg/kg) induced a clear antipsychotic-like effect, similar to the highest effective doses of VU152100 (Fig. 5b). The co-administration of 0.25 mg/kg VU152100 with active doses of LSP4-2022 or the co-administration of active doses of VU152100 (0.5 and 1 mg/kg) with three doses of LSP4-2022 (1, 2, and 4 mg/kg) reversed the action of MK-801 to the level achieved by the administration of the most active dose of VU152100 or LSP4-2022 alone (Fig. 5b). No enhancement of the activity of active doses of each compound was observed.

Neither VU152100 nor the combination of subeffective doses of VU152100 with LSP4-2022 changed the behavior of animals when administered without MK-801 (Table 3).

### DOI-induced spontaneous sEPSCs

Voltage-clamp recordings were obtained from layer V cortical cells in the presence of picrotoxin (30 μM), which blocks GABA_A_ receptor-mediated currents, to investigate the effects of DOI on sEPSCs. All recorded cells (*n* = 69) had electrophysiological characteristics of regular spiking pyramidal neurons (tested in current clamp; McCormick et al. 1985). Their mean resting membrane potential (RMP) was − 74 ± 5 mV and the mean input resistance (*R*_in_) was 252 ± 27 MΩ. The mean basal frequency of spontaneous synaptic activity ranged from 2.9 to 7.5 Hz (4.9 ± 0.3 Hz) and its mean amplitude was 9.77 ± 0.3 pA. sEPSCs were blocked by CNQX (5 μM), indicating that they were mediated by AMPA/kainate glutamate receptors (data not shown). The application of DOI (10 μM) systematically increased the mean sEPSC frequency, with an effect ranging from 127 ± 3.151 to 133 ± 3.312% of the control.

**Fig. 6:**
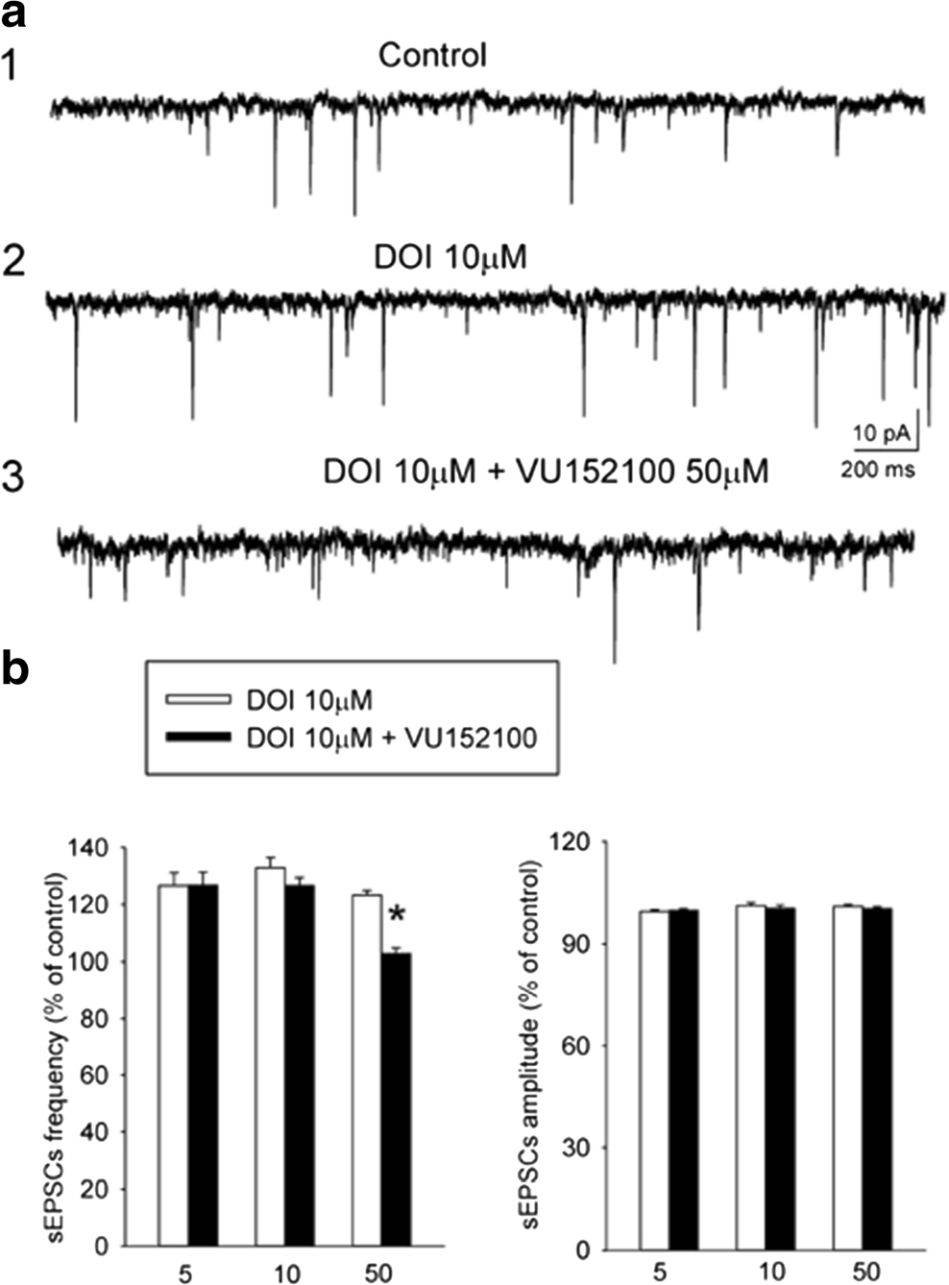
Effects of VU152100 on DOI-induced spontaneous EPSCs. **a** Examples of recordings from a representative neuron: (1) control activity, (2) recording obtained after a 10-min incubation with DOI, and (3) recording obtained after a 10-min incubation with VU152100 in the presence of DOI. **b** VU152100 (50 μM) suppressed the effect of DOI on the mean frequency of the sEPSCs. Data are presented as means ± SEM. Statistical analysis: *t* = 6.015; *df* = 9; *P < 0.0001 compared with the DOI-incubated group (concentration of VU152100 = 5 mM; *N* = 3, *n* = 11, 10 mM; *N* = 3, *n* = 9, 50 mM; *N* = 3, *n* = 10). *N* animal number, *n* cell number

**Fig. 7:**
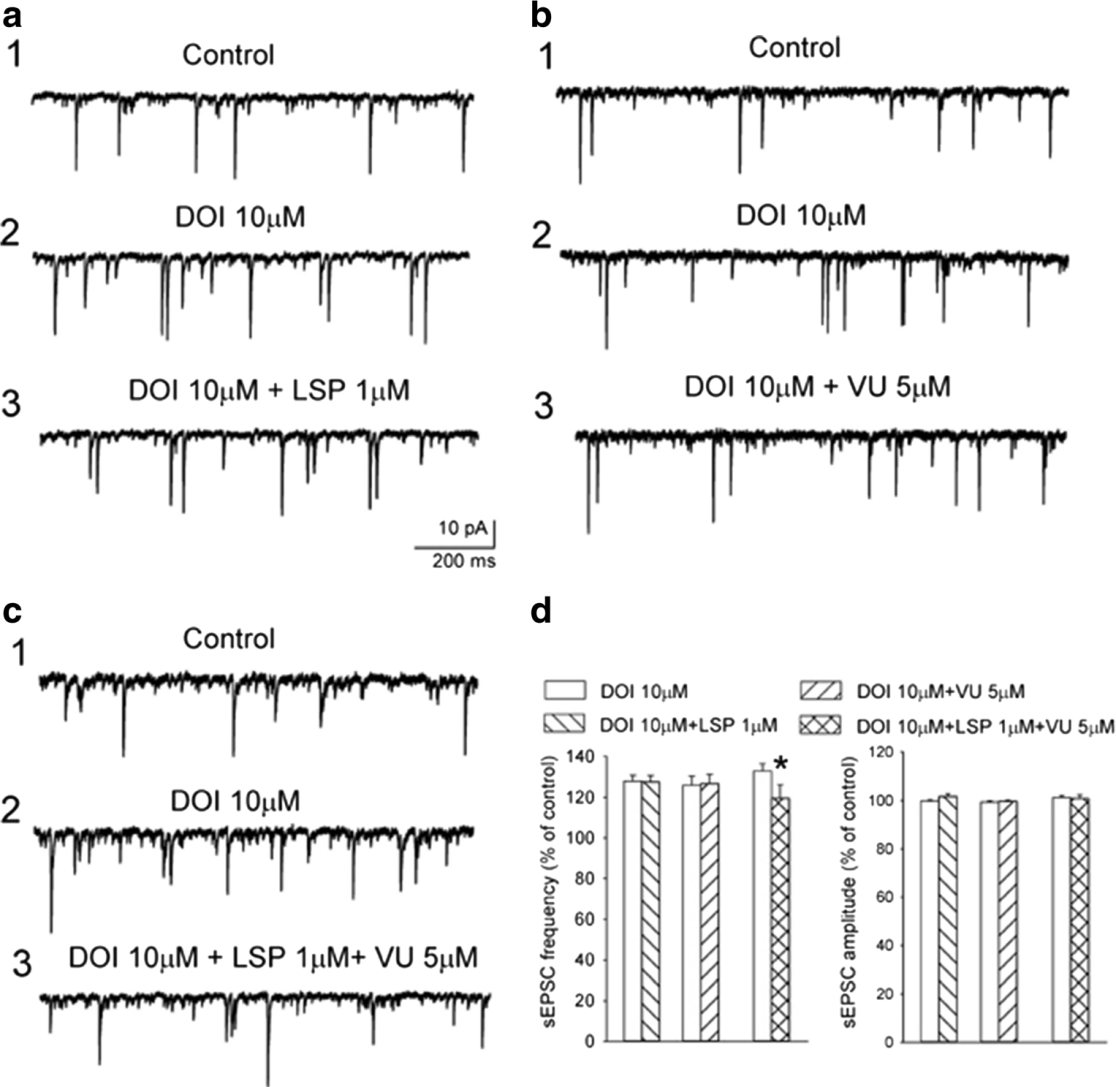
Effects of LSP4-2022 (LSP) and/or VU152100 (VU) administration on DOI-induced increase in sEPSC frequency. While 1 μM LSP (**a**) or 5 μM VU (**b**) applied alone does not change the effect of DOI, joint application of 1 μM LSP and 5 μM VU (**c**) results in a weakening of DOI-induced increase in sEPSC frequency. **d** Mean ± SEM sEPSC frequency and amplitude in all experimental groups. Statistical analysis: *N* = 3, *n* = 7; *t* = 6.16; *df* = 6; *P < 0.001, compared with a respective DOI-incubated cells. Labels in **a**–**c**: (1) control activity, (2) recording obtained after a 10-min incubation with DOI, and (3) recording obtained after a 10-min incubation with LSP and/or VU in the presence of DOI. Scale bars in **a** refer also to **b** and **c**. (LSP; *N* = 3, *n* = 7, VU152100; *N* = 3, *n* = 11, LSP+VU152100; *N* = 3, *n* = 7). *N* animal number, *n* cell number

Based on the measurements obtained from a separate group of five neurons, the effect of DOI on sEPSCs was not desensitized after 40 min of continuous application of DOI (Figs. 6a and 7a–c).

Three concentrations of VU152100 (5, 10m and 50 μM) were applied concurrently with DOI. The administration of 50 μM VU152100 reversibly suppressed the DOI-induced increase in the frequency but did not affect the mean amplitude of sEPSCs (*n* = 10; *t* = 6.015; *df* = 9; *P* < 0.0001) (Fig. 6b). LSP4-2022 when given in not effective dose (1 μM) together with non-effective dose of VU152100 (5 μM) significantly reversed the effect of DOI (*n* = 7; *t* = 6.16; *df* = 6; *P* < 0.001) (Fig. 7d).

### Amphetamine-induced hyperactivity

**Fig. 8:**
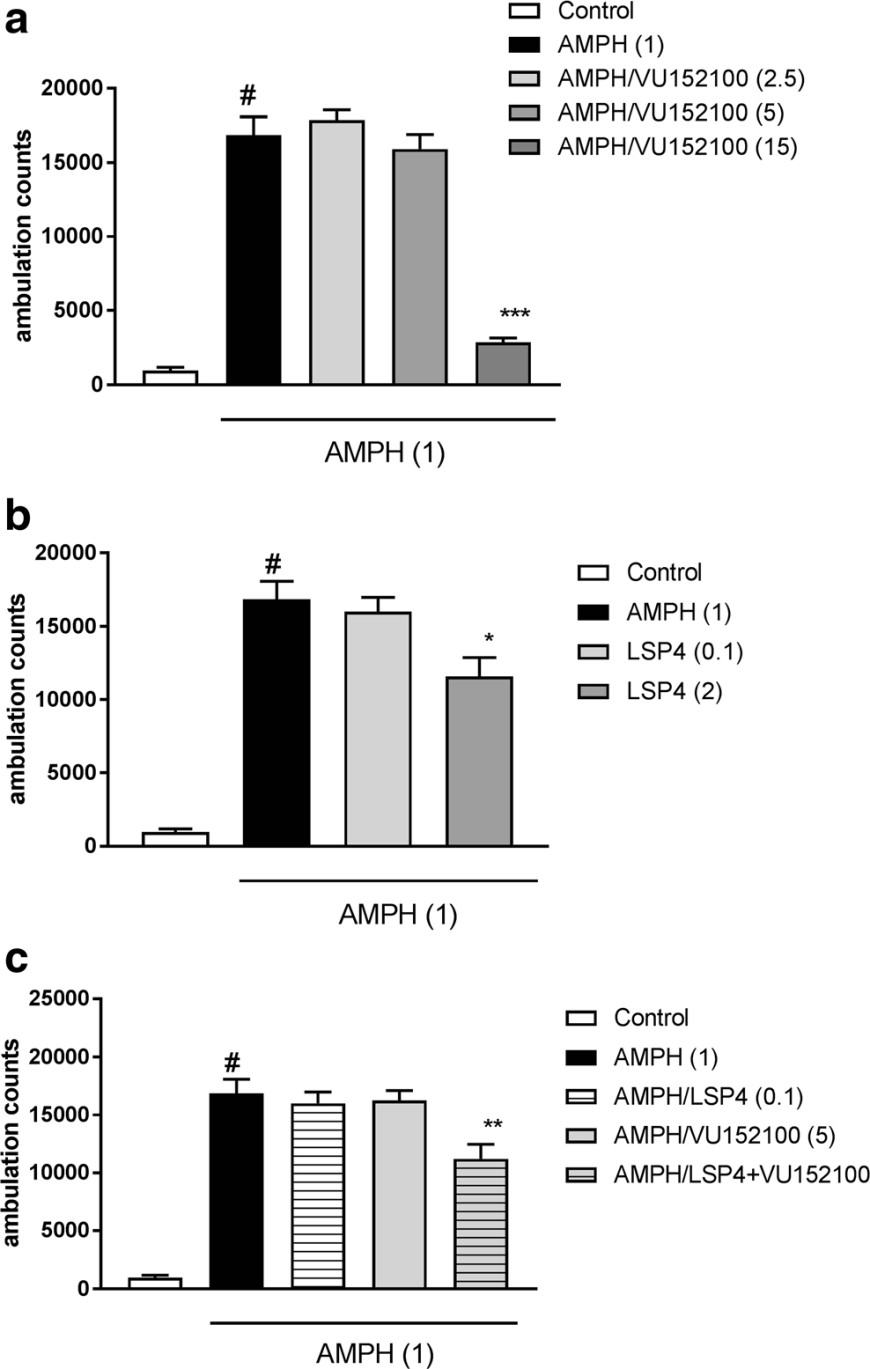
Effects of VU152100 (**a**), LSP4 (**b**), and the combined administration of VU152100 with LSP4 (**c**) on amphetamine-induced hyperactivity in rats that had been habituated to locomotor activity cages. LSP4 and VU152100 were administered 45 and 30 min, respectively, before amphetamine (AMPH) administration. Locomotor activity was measured for 60 min immediately after AMPH injection. Doses in milligrams per kilogram are indicated in parentheses. Data are presented as means ± SEM. One-way ANOVA [*F*_(3.30)_ = 54.65; *P* < 0.0001] (**a**) and [*F*_(2.25)_ = 5.74; *P* < 0.01] (**b**). Number of animals in groups *n* = 8–10. Two-way ANOVA of the effects of the interaction [*F*_(1.31)_ = 6.1; *P* < 0.02]. ^#^*P* < 0.001 compared with the control group, ***P < 0.0001, **P < 0.01, and *P < 0.05 compared with the AMPH-treated group. Number of animals in groups *n* = 8–10

The administration of a 1-mg/kg dose of amphetamine induced a robust increase in locomotor activity. VU152100 was injected at doses of 2.5, 5, and 15 mg/kg and reversed the action of amphetamine at the highest doses (Fig. 8a), whereas LSP4-2022 was effective at the dose of 2 mg/kg (Fig. 8b).

The co-administration of both compounds at subthreshold doses (LSP4-2022 0.1 mg/kg and VU152100 5 mg/kg) significantly reduced amphetamine-induced hyperactivity (Fig. 8c).

### Impact of LSP4-2022 and VU152100 on D_2_ receptor occupancy by [^18^F]fallypride measured using microPET

**Fig. 9:**
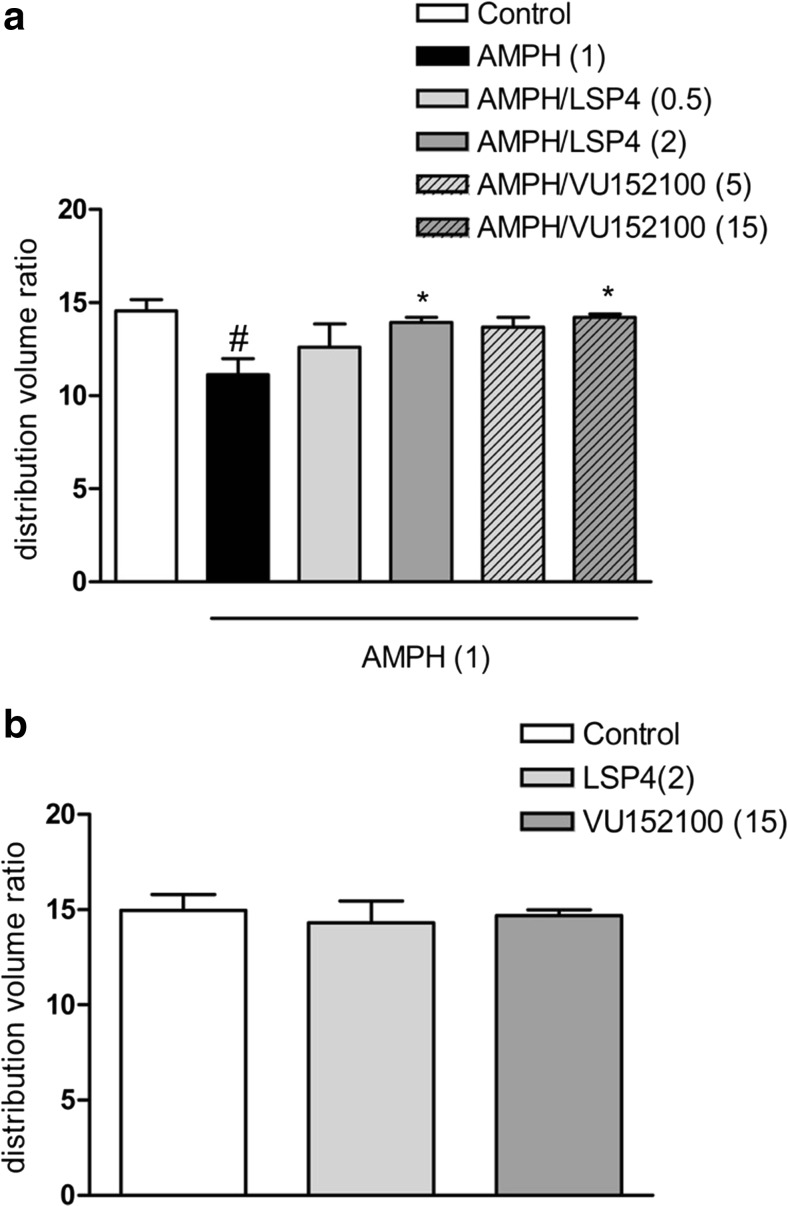
Distribution volume ratio (DVR′) estimates of rats injected with [^18^F]fallypride and imaged in the microPET for 60 min. Results are presented as means ± SEM. ^#^*P* < 0.005 compared with the controls and *P < 0.05 compared with the amphetamine-treated group. Data are presented as mean standard uptake values ± SEM. Number of animals in groups *n* = 6 except LSP4 (0.1) and VU (5) where *n* = 3

**Fig. 10:**
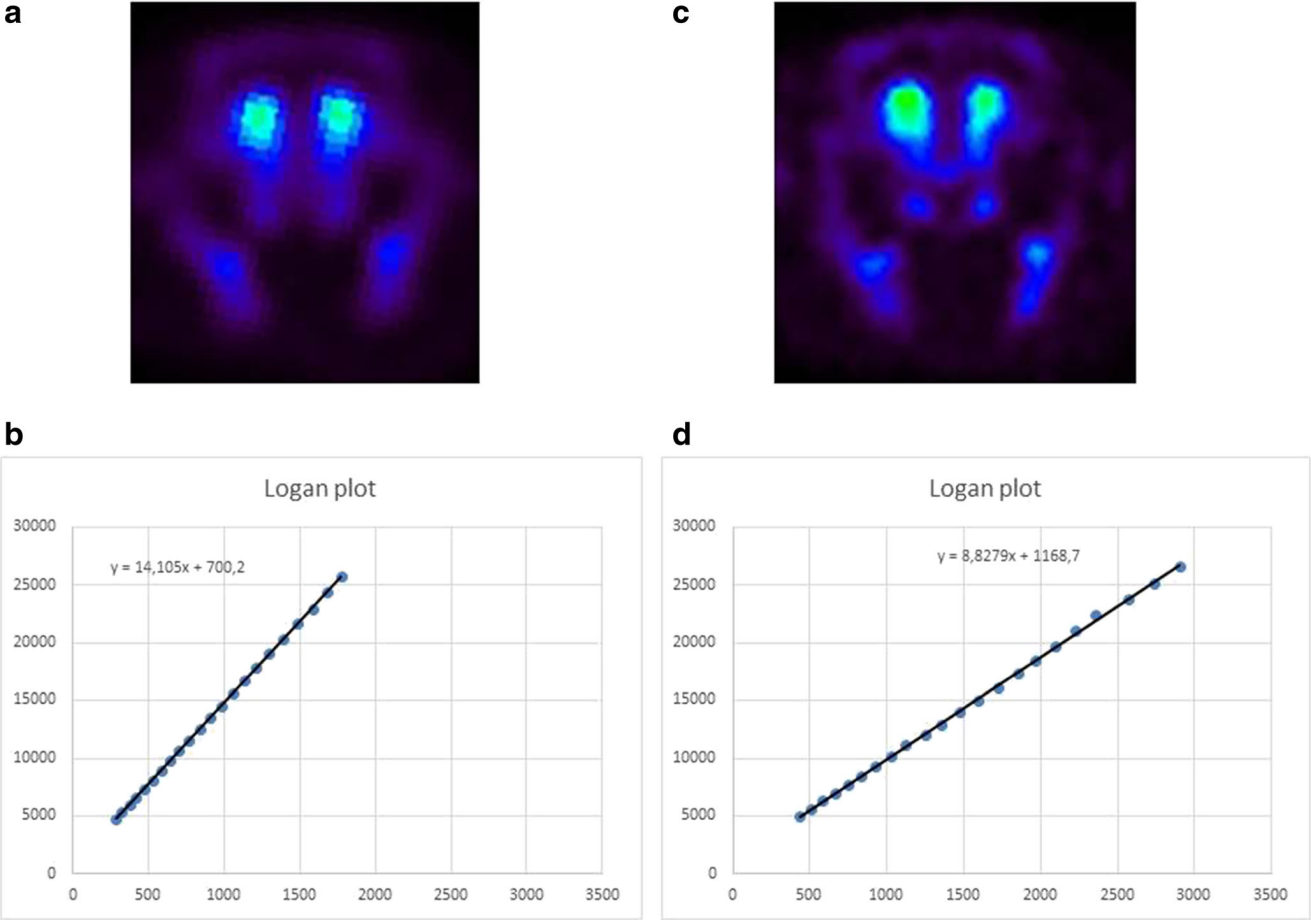
Representative positron emission tomography images of [^18^F]fallypride binding in vehicle- (**a**) and amphetamine-treated (**b**) rat brains. Representative Logan plots for vehicle- (**c**) and amphetamine-treated rats (**d**). The statistical analysis revealed *F*_(2.8)_ = 3.79; *P* < 0.05 for LSP4-2022 and *F*_(2.8)_ = 5.44; *P* < 0.05 for VU152100

The DVR′ measured in control rats was 14.56 ± 0.59. Amphetamine administration (1 mg/kg) induced a significant reduction in the DVR′ estimates of up to 23%, which was 11.14 ± 0.84 of control. LSP4-2022 and VU152100 reversed the amphetamine-induced effects at the highest doses (2 and 15 mg/kg, respectively) (Fig. 9a). These compounds did not have any effect on DVR′ estimates when administered alone (Fig. 9b). Representative images and Logan plots are shown in Fig. 10.

### Motor coordination

**Fig. 11:**
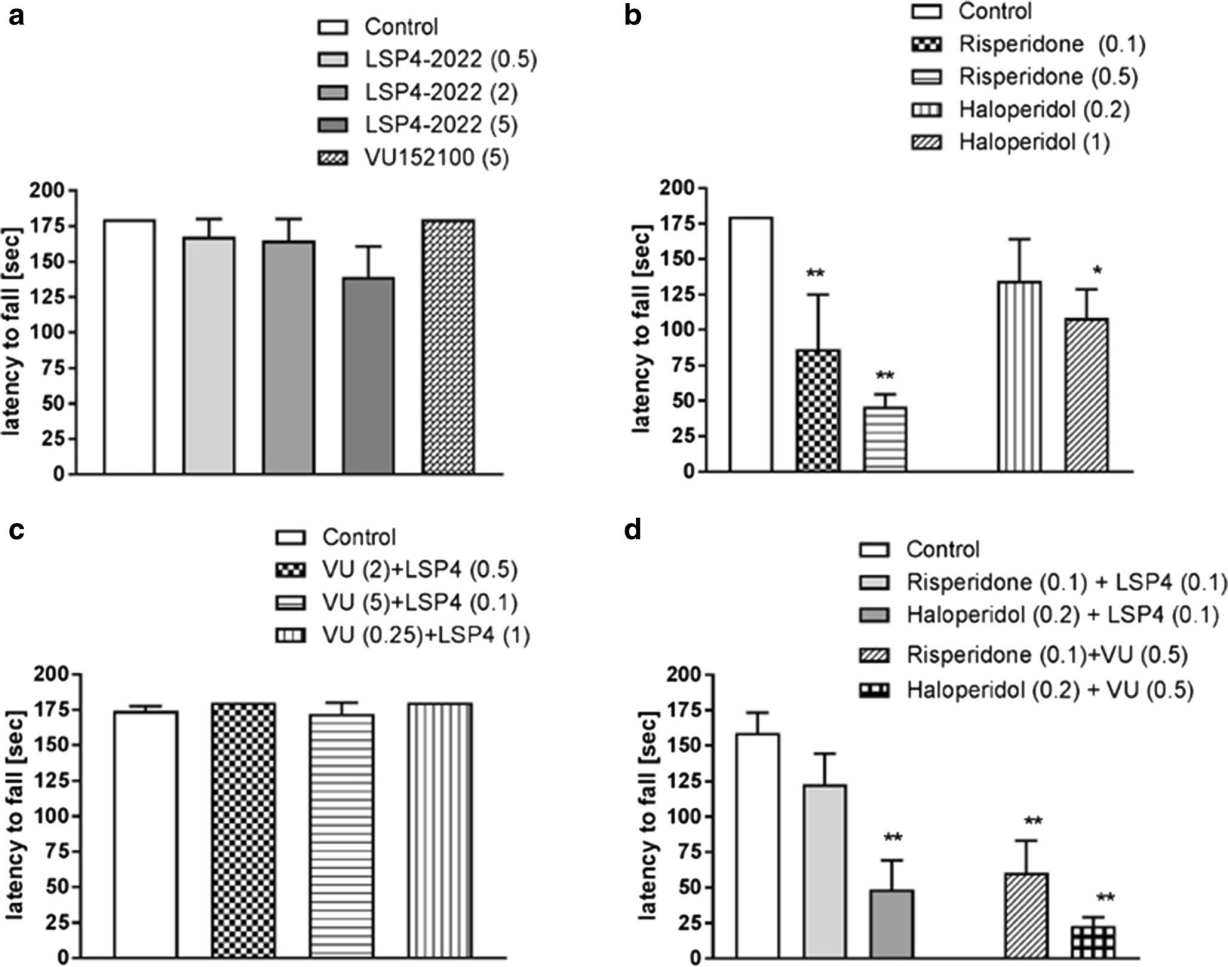
Effects of risperidone and haloperidol (**a**), LSP4-2022 (LSP4), and VU152100 (VU) (**b**) and the combination of subeffective doses of drugs together (**c**) or in the combination with standard neuroleptics (**d**) on rotarod performance in mice. Doses in milligrams per kilogram are indicated in parentheses. Data are presented as means ± SEM. One-way ANOVA revealed statistically significant effect of both doses of risperidone [*F*_(2.20)_ = 25.88, *P* < 0.01] and of haloperidol [*F*_(2.20)_ = 4.7, *P* < 0.05]. Number of animals in risperidone groups *n* = 5–6 and in controls and haloperidol 8–10. The effect of combined treatment of LSP4-2022 with risperidone or haloperidol also disturbed motor coordination [*F*_(2.23)_ = 9.37, *P < 0.05 and **P < 0.01], similarly as the combination of both neuroleptics with VU152100 [*F*_(2.24)_ = 19.16, *P* < 0.01]. Number of animals in groups *n* = 8–10

In the rotarod test, neither of tested drugs at any dose significantly influenced motor coordination of mice (Fig. 11a). Standard neuroleptics, risperidone (0.1 and 0.5 mg/kg) and haloperidol at the higher dose 1 mg/kg disturbed motor coordination of animals (Fig. 11b). The simultaneous administration of LSP4-2022 and VU152100 had no effect on the behavior of animals as well (Fig. 11c). However, the co-administration of both drugs in subeffective doses with subeffective dose of haloperidol (0.2) disturbed motor coordination in a statistically significant manner. The co-administration of subeffective dose of LSP4-2022 (0.1) with subeffective dose of haloperidol (0.2) also disturbed motor coordination, but such an effect was not observed when LSP4-2022 (0.1) was co-administered with subeffective dose of risperidone (0.1) (Fig. 11d).

## Discussion

In the present paper, the synergic/mutual interaction between muscarinic M_4_ and metabotropic glutamatergic mGlu_4_ receptors was examined in animal models of schizophrenia.

This is a follow-up study of our previous research on antipsychotic-like activity of mGlu_4_ receptor orthosteric agonists and PAMs (Wierońska et al. 2010, Wierońska et al. 2012, 2013, Woźniak et al. 2016, 2017). In this set of studies, mGlu_4_ agonist and M_4_ PAM were investigated. The subthreshold doses of the mGlu_4_ agonist LSP4-2022 and the M_4_ positive allosteric modulator VU152100 were administered simultaneously to investigate the putative mutual interaction between mGlu_4_ and M_4_ receptors. This combination exhibited efficacy similar to that observed for the administration of active doses of each compound alone in reversing hyperactivity in mice and rats, in a social interaction test, modified forced swim test, and in novel object recognition test. The effect observed in DOI-induced head twitches was clear but did not reach statistical significance. In social interaction and novel object recognition tests, we did more extensive research and each dose of VU152100 was co-administered with subtreshold and active dose of LSP4-2022, which were selected on the basics of our previous studies (Woźniak et al. 2017). The results indicate that only the simultaneous administration of subthreshold doses of both compounds reverses MK-801-induced deficits, and no enhancement of the activity of active doses was observed when they were co-administered with either active or subtreshold dose of the other compound.

There are some reports on the activity of each compound published so far. LSP4-2022, one of the best orthosteric agonists of the mGlu_4_ receptor, was previously used in our laboratory in both mice and rats in variety of behavioral and neurochemical studies (Woźniak et al. 2016, 2017). VU152100 was introduced in 2008 (Brady et al. 2008) and is one of the two commercially available selective M_4_ positive allosteric modulators. All behavioral studies that have been published with this compound have predominantly been performed in rats and were performed in dopaminomimetic-based animal models (Brady et al. 2008; Byun et al. 2014; Dencker et al. 2012; Galloway et al. 2014). No published studies have examined the activity of the compound in MK-801-based animal models of schizophrenia, although the administration of an NMDA antagonist better resembles schizophrenia arousal than dopaminomimetics (Javitt 2004; Conn et al. 2009c; Moghaddam and Jackson 2003, 2012). Therefore, in the present research, dose dependence studies showing activity of the compound in MK-801-based models of negative and cognitive symptoms of schizophrenia were carried out for the first time. In our experiments, lower doses of VU152100 were active compared to the results of these earlier reports.

In the second part of the studies, selected actions of VU152100 and LSP4-2022 on glutamatergic and dopaminergic neurotransmission were investigated, using patch-clamp recordings and PET imaging studies.

Earlier, it was shown that LSP4-2022 reversed DOI-induced increases in both frequency and the amplitude of spontaneous EPSCs, confirming its ability to restore DOI-induced increases in glutamatergic system activity (Woźniak et al. 2017). Here we show that VU152100 attenuates the increase of sEPSC frequency triggered by DOI (via activation of postsynaptic 5-HT_2A/2C_ receptors) in layer V pyramidal neurons in cortical slices. Similar effect was observed when both compounds were applied simultaneously at the subthreshold doses. The result is in line with behavioral observation, in which simultaneous action of both ligands was also observed, although in not statistical manner. The attenuation of glutamate-induced sEPSC frequency indicates that the compounds exert their action via presynaptic mechanism (van der Kloot 1991). Previously, in the paper of Pancani et al., it was shown that VU152100 potentiated CCh-induced depression of EPSCs via an increase in paired pulse ratio, thereby indicating that M_4_-mediated depression of EPSCs in medium spiny neurons (more than 95% of all striatal neuronal population (Kreitzer 2009)) is probably due to decrease in presynaptic glutamate release (Pancani et al. 2014). Comparing to this paper, in our studies, much higher dose of the compound was needed to inhibit DOI-induced sEPSCs in the cortical slices.

Considering the mechanism by which VU152100 exerts its action on DOI-induced head twitches, it should be mentioned that cholinergic interneurons exert powerful modulation of circuit activity within the brain, and M_4_ receptors expressed on their terminals play essential role in the regulation of acetylcholine release. This released acetylcholine can reciprocally activate dopaminergic neuronal activity via nicotinic receptors, and both agonists and antagonists of nicotinic receptors reverse DOI-induced head twitches (Tizabi et al. 2001). Therefore, the precise mechanism by which cholinergic system is involved in the inhibition of DOI-induced head twitches, except corticostriatal transmission, is yet to be established.

Subsequently, the effects of VU152100 and LSP4-2022 were investigated on D_2_ receptors in the striatum with PET imaging studies (Tantawy et al. 2009, 2011). Amphetamine administration increased dopamine release and subsequently increased the occupancy of D_2_ receptors in the striatum, thereby reducing the number of unoccupied D_2_ receptors (Tantawy et al. 2009, 2011). Active doses of both investigated compounds reversed this amphetamine-induced effect. The fact that both drugs with nondopaminergic mechanism of action are able to restore amphetamine-induced changes in striatum seems to be of importance, as for years, the hyperactivity of the dopaminergic system in the striatum has been regarded as the primary factor triggering the onset of positive symptoms of schizophrenia (Haracz 1982; Heinz and Schlagenhauf 2010). Therefore, the reversal of dopaminergic dysfunction in this structure is crucial for antipsychotic efficacy. However, chronic blockade of D_2_ receptors in the striatum contributes to the development of adverse effects observed after standard neuroleptics. Thus, compounds that inhibit glutamatergic and dopaminergic neurotransmission without direct blockade of D_2_ receptors are desired as novel antipsychotics (Fervaha et al. 2015, 2016). It seems that both M_4_ and mGlu_4_ PAMs fulfill these criteria and may not induce adverse effects typical for standard neuroleptics. Earlier it was shown that both compounds reversed haloperidol-induced catalepsy and/or did not induce catalepsy by themselves (Goudet et al. 2012; Byun et al. 2014). Here we used rotarod test to establish if the compounds influence motor coordination in animals. Neither LSP4-2022 nor VU152100 impaired balance and motor coordination when administered at the doses higher than those that were effective in behavioral studies. Also the combinations of the compounds in subtreshold doses did not influence the rotarod performance. Standard neuroleptics (haloperidol, risperidone) impaired rotarod performance and the administration of low/subtreshold doses of those neuroleptics with subtreshold doses of VU0152100 or LSP4-2022 also affected motor coordination in mice. It should be mentioned that performance on the rotarod allows assessing one aspect of antipsychotic-induced adverse effects. However, the most relevant measures on long-term treatment with neuroleptics are tardive dyskinesia (involuntary, repetitive body movements, such as grimacing, sticking out the tongue, or smacking of the lips) which results primarily from neuroleptic-induced dopamine supersensitivity in the nigrostriatal pathway, with the D_2_ dopamine receptor being most affected (Carbon et al. 2017). Therefore, it seems that the optimal pharmacological interventions in schizophrenic patients should omit direct blockade of dopaminergic receptors in the striatum. Simultaneous administration of M_4_/mGlu_4_ receptors can be proposed as one of the directions. Except the activity of the ligands on glutamatergic and/or dopaminergic system presented here, it was also shown that their administration reduced amphetamine or MK-801-induced dopamine release in the striatum or prefrontal cortex (Byun et al. 2014; Woźniak et al. 2017).

Neither of the drugs that are approved and currently used in the clinic stimulates mGlu_4_ and/or M_4_ receptors. We propose to combine the two ligands and minimize the doses used to reduce the risk of overdosing and omitting putative adverse effects that could develop. Both receptors investigated here are coupled to G_o/i_ signaling and are expressed in the brain circuits involved in schizophrenia, including the striatum, cortex, and hippocampus (Hersch et al. 1994; Levey et al. 1991, 1995). The M_4_ receptor regulates the activity of dopaminergic and/or acetylcholinergic neurons in the striatum and nucleus accumbens (Ince et al. 1997; Jeon et al. 2010; Dencker et al. 2012; Nadal et al. 2016; Pancani et al. 2014; Bell et al. 2013; Kuroiwa et al. 2012). Similar action may exert mGlu_4_ receptors (Pancani et al. 2014). Therefore, the ligands may complement each other’s action and putatively be active in subjects with lower expression of mGlu_4_ and /or M_4_ receptors or in subjects with partially impaired function of those receptors.

## Electronic supplementary material

ESM 1 (PDF 815 kb)

## Abbreviations

DALYs: Disability adjusted life years
CNS: Central nervous system
mGlu: Metabotropic glutamate
PAM: Positive allosteric modulator
Ach: Acetylcholine
M: Muscarinic
PET: Positron emission tomography
KO: Knockout
NOR: Novel object recognition
DVR: Distribution volume ratio
ROI: Region of interest
