# Supplementary material for: Mutual activation of glutamatergic mGlu4 and muscarinic M4 receptors reverses schizophrenia-related changes in rodents
Source: Psychopharmacology (Berl). 2018 Jul 27;235(10):2897–913. doi: 10.1007/s00213-018-4980-y (PMC6182605; doi:10.1007/s00213-018-4980-y)
Supplement: Supplementary file 1 — (PDF 815 kb) [file 213_2018_4980_MOESM1_ESM.pdf]

Study Report

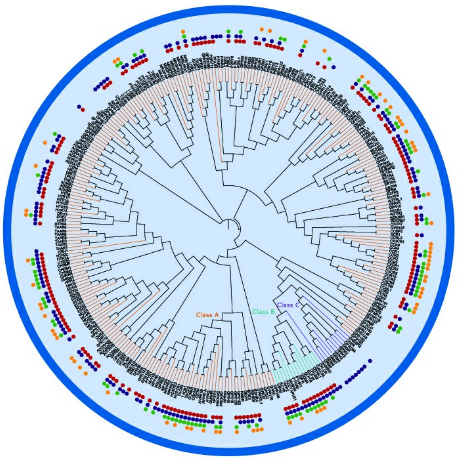

Requester(s): Joanna M Wieronska

Company: Department of Neurobiology - Inst. of Pharm. PAS

Report Date: 1/18/2018

Quote ID: GPCRC10414D

Order ID: POS001

Service: gpcrSCAN

Number of Compounds Tested: 1

Number of Assays Tested: 5

Director, Profiling Services: Dr. Neil Charter

Phone: (510) 771-3542

Project Manager: Sharon Irelan / Melinda Stampfl

Phone: (858) 224-6925 / (858) 224-6933

www.services.discoverx.com

DiscoverX Corporation

42501 Albrae Street, Suit 100

Fremont, CA 94538

DiscoverX LeadHunter DISCOVERY SERVICES

Customer Information

Company : Department of Neurobiology - Inst. of Pharm. PAS

Client Name : Joanna M Wieronska

Address : Smetna 12

Kraków, Poland 31-343

Email : wierons@if-pan.krakow.pl

Report Summary

Assays : **5**

Compounds : **1**

Objective : **Agonist Primary Screen**

Summary : **DiscoverX successfully profiled 1 compound with 5 Biosensor Assays.**

The data is provided on the result page. The data is also provided in the an accompanying spreadsheet file.

Technology Principle

Arrestin Pathway

The PathHunter® β-Arrestin assay monitors the activation of a GPCR in a homogenous, non-imaging assay format using a technology developed by DiscoverX called Enzyme Fragment Complementation (EFC) with β-galactosidase (β-Gal) as the functional reporter. The enzyme is split into two inactive complementary portions (EA for Enzyme Acceptor and PK for ProLink) expressed as fusion proteins in the cell. EA is fused to β-Arrestin and PK is fused to the GPCR of interest.

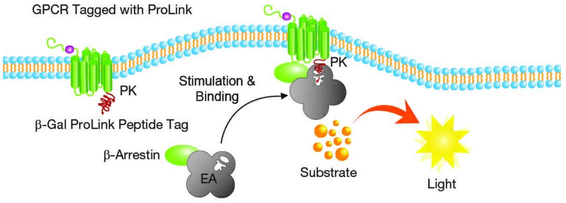

When the GPCR is activated and β-Arrestin is recruited to the receptor, ED and EA complementation occurs, restoring β-Gal activity which is measured using chemiluminescent PathHunter® Detection Reagents.

Endocytosis Pathway

Using EFC technology, DiscoverX has developed several methods to study receptor internalization.

PathHunter® Activated GPCR Internalization Assays provide a quantitative measurement of arrestin-mediated GPCR internalization, allowing you to monitor the movement of unlabeled, arrestin-bound GPCRs from the plasma membrane in live cells. In this system, EA is fused to arrestin (EA-Arrestin) and ED is localized exclusively to the surface of early endosomes. Enzyme activity is restored upon GPCR activation and arrestin-mediated trafficking to early endosomes. Activity is measured using PathHunter® Detection Reagents.

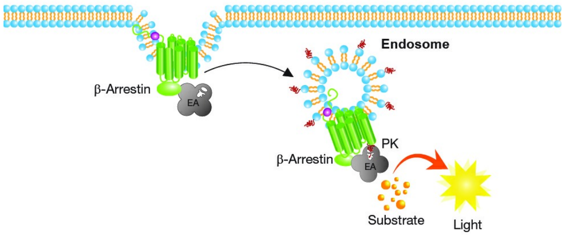

PathHunter® Total GPCR Internalization Assays provide a quantitative measurement of total GPCR protein internalized into endosomes and is measured using PathHunter® Detection Reagents.

There are two Total GPCR Internalization assay formats. In the first, one of the EFC components is localized exclusively to the endosome and the other component is fused to the GPCR of interest.

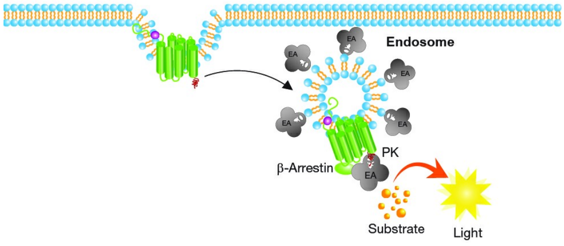

When stimulation of the target receptor results in receptor internalization and trafficking to early endosomes, complementation of the two enzyme fragments occurs, reflected as an increase in enzyme activity.

In the second format, EA is localized exclusively to the plasma membrane (EA-Membrane) and ED is fused to the GPCR of interest. Membrane-bound receptors will complement with EA, resulting in high levels of enzyme activity. When activation of the GPCR results in receptor internalization, loss of receptor at the cell surface is reflected as a loss of enzyme activity.

cAMP Secondary Messenger Pathway

DiscoverX has developed a panel of cell lines stably expressing non-tagged GPCRs that signal through cAMP. Hit Hunter® cAMP assays monitor the activation of a GPCR via Gi and Gs secondary messenger signaling in a homogenous, non-imaging assay format using a technology developed by DiscoverX called Enzyme Fragment Complementation (EFC) with β-galactosidase (β-Gal) as the functional reporter.

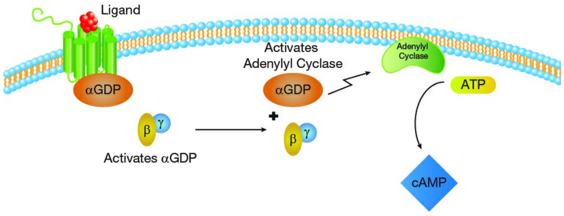

The enzyme is split into two complementary portions: EA for Enzyme Acceptor and ED for Enzyme Donor. ED is fused to cAMP and in the assay competes with cAMP generated by cells for binding to a cAMP-specific antibody. Active β-Gal is formed by complementation of exogenous EA to any unbound ED-cAMP. Active enzyme can then convert a chemiluminescent substrate, generating an output signal detectable on a standard microplate reader.

Calcium Secondary Messenger Pathway

The Calcium No Wash^{PLUS} assay monitors the activation of a GPCR via Gq secondary messenger signaling in a live cell, non-imaging assay format.

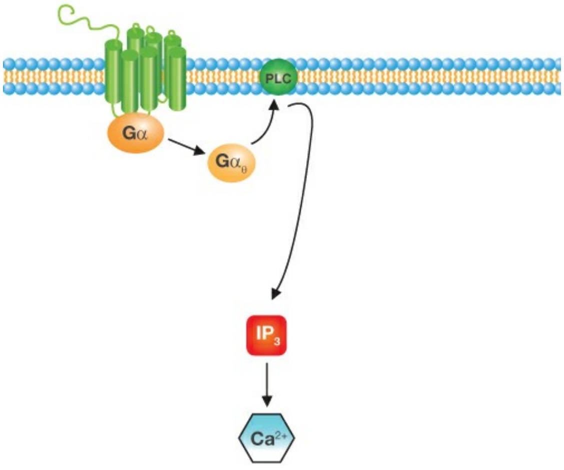

Calcium mobilization in PathHunter® cell lines or other cell lines stably expressing Gq-coupled GPCRs is monitored using a calcium-sensitive dye that is loaded into cells. GPCR activation by a compound results in the release of calcium from intracellular stores and an increase in dye fluorescence that is measured in real-time.

Profile Overview

DiscoverX was contracted by Dr. Joanna M Wieronska at Department of Neurobiology - Inst. of Pharm. PAS to profile 1 compound with 5 GPCR biosensor assays. Details are given below:

Project Description

Product:	GPCR
Assays:	5
# Compound(s):	1
# Datapoints:	10
Targets:	CHRM1 - Arrestin - Agonist<br>CHRM2 - Arrestin - Agonist<br>CHRM3 - Arrestin - Agonist<br>CHRM4 - Arrestin - Agonist<br>CHRM5 - Arrestin - Agonist

Assay Design: GPCR Arrestin

Cell Handling

1. PathHunter cell lines were expanded from freezer stocks according to standard procedures.
2. Cells were seeded in a total volume of 20 µL into white walled, 384-well microplates and incubated at 37°C for the appropriate time prior to testing.

Agonist Format

1. For agonist determination, cells were incubated with sample to induce response.
2. Intermediate dilution of sample stocks was performed to generate 5X sample in assay buffer.
3. 5 µL of 5X sample was added to cells and incubated at 37°C or room temperature for 90 to 180 minutes. Vehicle concentration was 1%.

Inverse Agonist Format

1. For inverse agonist determination, cells were incubated with sample to induce response.
2. Intermediate dilution of sample stocks was performed to generate 5X sample in assay buffer.
3. 5 µL of 5X sample was added to cells and incubated at 37°C or room temperature for 3 to 5 hours. Vehicle concentration was 1%. Extended incubation is typically required to observe an inverse agonist response in the PathHunter arrestin assay.

Positive Allosteric Modulation Format

1. For allosteric determination, cells were pre-incubated with sample followed by agonist induction at the EC20 concentration.
2. Intermediate dilution of sample stocks was performed to generate 5X sample in assay buffer.
3. 5 µL of 5x sample was added to cells and incubated at 37°C or room temperature for 30 minutes. Vehicle concentration was 1%.
4. 5 µL of 6X EC20 agonist in assay buffer was added to the cells and incubated at 37°C or room temperature for 90 or 180 minutes.

Antagonist/Negative Allosteric Modulation Format

1. For antagonist determination, cells were pre-incubated with antagonist followed by agonist challenge at the EC80 concentration.
2. Intermediate dilution of sample stocks was performed to generate 5X sample in assay buffer.
3. 5 µL of 5x sample was added to cells and incubated at 37°C or room temperature for 30 minutes. Vehicle concentration was 1%.
4. 5 µL of 6X EC80 agonist in assay buffer was added to the cells and incubated at 37°C or room temperature for 90 or 180 minutes.

Signal Detection

1. Assay signal was generated through a single addition of 12.5 or 15 µL (50% v/v) of PathHunter Detection reagent cocktail, followed by a one hour incubation at room temperature.
2. Microplates were read following signal generation with a PerkinElmer Envision™ instrument for chemiluminescent signal detection.

Data Analysis

1. Compound activity was analyzed using CBIS data analysis suite (ChemInnovation, CA).
2. For agonist mode assays, percentage activity was calculated using the following formula:

   % Activity =100% x (mean RLU of test sample - mean RLU of vehicle control) / (mean MAX control ligand - mean RLU of vehicle control).

3. For inverse agonist mode assays, percentage activity was calculated using the following formula:

   % Inverse Agonist Activity =100% x (1 - (mean RLU of test sample - mean RLU of vehicle control) / (mean RLU of vehicle control)).

4. For positive allosteric mode assays, percentage modulation was calculated using the following formula:

   % Modulation =100% x ((mean RLU of test sample - mean RLU of EC20 control) / (mean RLU of MAX control ligand - mean RLU of EC20 control)).

5. For antagonist and negative allosteric mode assays, percentage inhibition was calculated using the following formula:

   % Inhibition =100% x (1 - (mean RLU of test sample - mean RLU of vehicle control) / (mean RLU of EC80 control - mean RLU of vehicle control)).

Results:

Compound Name	Project ID	Assay Name	Assay Format	Assay Target	Result Type	RC50 (uM)	Hill	Curve Bottom	Curve Top	Max Response	Result Graph
Acetylcholine	POS001-01-c-00001-000-00	Arrestin	Agonist	CHRM1	EC50	1.777203	0.61415	0	98.23	99.372	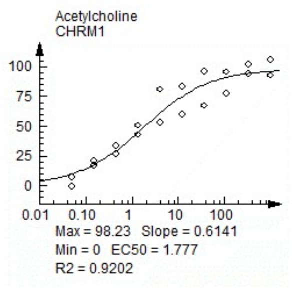
Acetylcholine	POS001-01-c-00001-000-00	Arrestin	Agonist	CHRM2	EC50	16.68783	1.086	0.56372	99.828	101.6	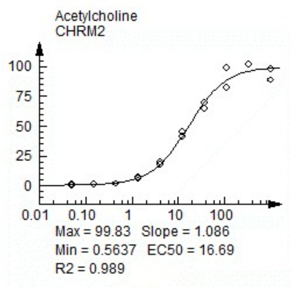
Acetylcholine	POS001-01-c-00001-000-00	Arrestin	Agonist	CHRM3	EC50	0.9840742	0.79556	0.097756	96.209	100.11	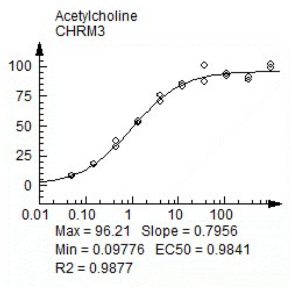
Acetylcholine	POS001-01-c-00001-000-00	Arrestin	Agonist	CHRM4	EC50	0.5793233	1.2644	0	85.511	92.179	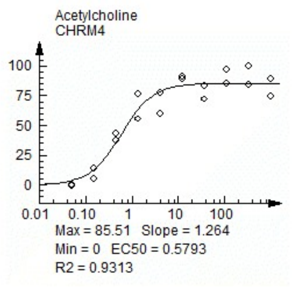
Acetylcholine	POS001-01-c-00001-000-00	Arrestin	Agonist	CHRM5	EC50	0.1897903	0.56409	0	100	100.68	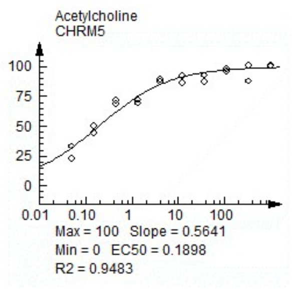

Figure 1: Control dose response curves for the selected GPCR Biosensor Assays

Control dose curves were performed for the requested GPCR Biosensor Assays. Data shown was normalized to the maximal and minimal response observed in the presence of control compound and vehicle respectively.

Results:

Compound Name	Project ID	Assay Name	Assay Format	Assay Target	Conc (uM)	Value 1	Value 2	Average Value	Std Deviation	% Efficacy
NB	POS001-01-c-00001-000-00	Arrestin	Agonist	CHRM1	1	808080	848400	828240	28510.5	-2.5
NB	POS001-01-c-00001-000-00	Arrestin	Agonist	CHRM1	10	763560	794640	779100	21976.9	-6
NB	POS001-01-c-00001-000-00	Arrestin	Agonist	CHRM1	25	790160	797440	793800	5147.7	-5
NB	POS001-01-c-00001-000-00	Arrestin	Agonist	CHRM1	50	797160	842240	819700	31876.4	-3.1
NB	POS001-01-c-00001-000-00	Arrestin	Agonist	CHRM1	100	800520	815080	807800	10295.5	-4
NB	POS001-01-c-00001-000-00	Arrestin	Agonist	CHRM2	1	68600	73920	71260	3761.8	2
NB	POS001-01-c-00001-000-00	Arrestin	Agonist	CHRM2	10	59360	67760	63560	5939.7	1.1
NB	POS001-01-c-00001-000-00	Arrestin	Agonist	CHRM2	25	57120	73640	65380	11681.4	1.3
NB	POS001-01-c-00001-000-00	Arrestin	Agonist	CHRM2	50	58240	60480	59360	1583.9	0.6
NB	POS001-01-c-00001-000-00	Arrestin	Agonist	CHRM2	100	54600	61040	57820	4553.8	0.4
NB	POS001-01-c-00001-000-00	Arrestin	Agonist	CHRM3	1	28840	33600	31220	3365.8	-2.8
NB	POS001-01-c-00001-000-00	Arrestin	Agonist	CHRM3	10	28000	31920	29960	2771.9	-3.1
NB	POS001-01-c-00001-000-00	Arrestin	Agonist	CHRM3	25	30520	30800	30660	198	-2.9
NB	POS001-01-c-00001-000-00	Arrestin	Agonist	CHRM3	50	29960	32480	31220	1781.9	-2.8
NB	POS001-01-c-00001-000-00	Arrestin	Agonist	CHRM3	100	33320	33880	33600	396	-2.1
NB	POS001-01-c-00001-000-00	Arrestin	Agonist	CHRM4	1	14560	16520	15540	1385.9	-7.7
NB	POS001-01-c-00001-000-00	Arrestin	Agonist	CHRM4	10	14280	19320	16800	3563.8	-2.7
NB	POS001-01-c-00001-000-00	Arrestin	Agonist	CHRM4	25	15680	16520	16100	594	-5.5
NB	POS001-01-c-00001-000-00	Arrestin	Agonist	CHRM4	50	16240	16800	16520	396	-3.8
NB	POS001-01-c-00001-000-00	Arrestin	Agonist	CHRM4	100	14560	15400	14980	594	-9.8
NB	POS001-01-c-00001-000-00	Arrestin	Agonist	CHRM5	1	1880760	1900640	1890700	14057.3	-8.1
NB	POS001-01-c-00001-000-00	Arrestin	Agonist	CHRM5	10	1920520	1965040	1942780	31480.4	-6.1
NB	POS001-01-c-00001-000-00	Arrestin	Agonist	CHRM5	25	1817200	1953280	1885240	96223.1	-8.3
NB	POS001-01-c-00001-000-00	Arrestin	Agonist	CHRM5	50	1838200	1966440	1902320	90679.4	-7.6
NB	POS001-01-c-00001-000-00	Arrestin	Agonist	CHRM5	100	1893360	1900080	1896720	4751.8	-7.9

Table 1: Compound activity with the GPCR Biosensor Assays

Compound was tested in agonist mode with the GPCR Biosensor Assays. For agonist assays, data was normalized to the maximal and minimal response observed in the presence of control ligand and vehicle.

Summary:

DiscoverX successfully profiled 1 compound with 5 Biosensor Assays.

The data is also provided in an accompanying spreadsheet file.

This is to certify that the data contained within this report was conducted as described above.

Dr. N. W. Charter

Director, Profiling Services
